# Nitrogen-induced terrestrial eutrophication: cascading effects and impacts on ecosystem services

**DOI:** 10.1002/ecs2.1877

**Published:** 2017-07-31

**Authors:** CHRISTOPHER M. CLARK, MICHAEL D. BELL, JAMES W. BOYD, JANA E. COMPTON, ERIC A. DAVIDSON, CHRISTINE DAVIS, MARK E. FENN, LINDA GEISER, LAURENCE JONES, TAMARA F. BLETT

**Affiliations:** 1 National Center for Environmental Assessment, Office of Research and Development, U.S. EPA, Washington, D.C. 20460 USA; 2 Air Resources Division, National Park Service, Lakewood, Colorado 80225 USA; 3 Resources for the Future, Washington, D.C. 20036 USA; 4 Western Ecology Division, Office of Research and Development, U.S. EPA, Corvallis, Oregon 97333 USA; 5 Appalachian Laboratory, University of Maryland Center for Environmental Science, Frostburg, Maryland 21532 USA; 6 Office of Air and Radiation, Office of Air Quality Planning and Standards, U.S. EPA, Research Triangle Park, North Carolina 27709 USA; 7 Pacific Southwest Research Station, USDA Forest Service, Riverside, California 92607 USA; 8 Washington Office-Water Wildlife Fish Air and Rare Plants, USDA Forest Service, Washington, D.C. 20250 USA; 9 Environment Centre Wales, Centre for Ecology and Hydrology, Deiniol Road, Bangor, LL57 2UW United Kingdom

**Keywords:** atmospheric deposition, critical loads, ecosystem services, Final Ecosystem Goods and Services, Special Feature, Air Quality and Ecosystem Services

## Abstract

Human activity has significantly increased the deposition of nitrogen (N) on terrestrial ecosystems over pre-industrial levels leading to a multitude of effects including losses of biodiversity, changes in ecosystem functioning, and impacts on human well-being. It is challenging to explicitly link the level of deposition on an ecosystem to the cascade of ecological effects triggered and ecosystem services affected, because of the multitude of possible pathways in the N cascade. To address this challenge, we report on the activities of an expert workshop to synthesize information on N-induced terrestrial eutrophication from the published literature and to link critical load exceedances with human beneficiaries by using the STressor–Ecological Production function–final ecosystem Services Framework and the Final Ecosystem Goods and Services Classification System (FEGS-CS). We found 21 N critical loads were triggered by N deposition (ranging from 2 to 39 kg N·ha^−1^·yr^−1^), which cascaded to distinct beneficiary types through 582 individual pathways in the five ecoregions examined (Eastern Temperate Forests, Marine West Coast Forests, North-western Forested Mountains, North American Deserts, Mediterranean California). These exceedances ultimately affected 66 FEGS across a range of final ecosystem service categories (21 categories, e.g., changes in timber production, fire regimes, and native plant and animal communities) and 198 regional human beneficiaries of different types. Several different biological indicators were triggered in different ecosystems, including grasses and/or forbs (33% of all pathways), mycorrhizal communities (22%), tree species (21%), and lichen biodiversity (11%). Ecoregions with higher deposition rates for longer periods tended to have more numerous and varied ecological impacts (e.g., Eastern Temperate Forests, eight biological indicators) as opposed to other ecoregions (e.g., North American Deserts and Marine West Coast Forests each with one biological indicator). Nonetheless, although ecoregions differed by ecological effects from terrestrial eutrophication, the number of FEGS and beneficiaries impacted was similar across ecoregions. We found that terrestrial eutrophication affected all ecosystems examined, demonstrating the widespread nature of terrestrial eutrophication nationally. These results highlight which people and ecosystems are most affected according to present knowledge, and identify key uncertainties and knowledge gaps to be filled by future research.

## Introduction

Human activity has increased the deposition of nitrogen (N) by 10-fold or more over pre-industrial levels for much of the developed world (Vitousek et al. 1997, Galloway et al. 2004, 2008). Intentional inputs of N in the form of fertilizer application to crops have been a boon to mankind, partially responsible for supporting global population increases to over 7 billion. In many areas of the globe, increased fertilizer N is still needed to improve agricultural output (Vitousek et al. 2009, Zhang et al. 2015). However, unintentional N enrichment primarily from fossil fuel combustion and losses from industrial agriculture has a variety of negative environmental impacts, including reductions in biodiversity (Stevens et al. 2010, Simkin et al. 2016), increased nutrient runoff to waterways (Stets et al. 2015), increased nitrate in groundwater (Nolan and Stoner 2000), hypoxia in coastal and inland waters (Scavia et al. 2003, Hagy et al. 2004), increased air pollution (EPA 2010), soil acidification (Driscoll et al. 2003, Sullivan et al. 2013), and alterations to global carbon and climate processes (Zaehle et al. 2010a, Pinder et al. 2012, USGCRP 2014) Each of these effects ultimately can impact ecosystem services and human wellbeing (Compton et al. 2011, Jones et al. 2014). In the eastern United States, N deposition is declining from historical peaks in the 1970s and 1980s as a result of stricter air quality standards associated with the Clean Air Act of 1990 and subsequent policy (Burns et al. 2011). However, deposition is increasing or unchanged in the western United States, and there is a shift in the composition of deposition toward more reduced forms of N nationally (Li et al. 2016). Further-more, even though deposition may be declining in some regions such as the east, these rates still far exceed pre-industrial rates and the estimated sensitivities of many ecological endpoints in the region (Baron et al. 2011, Pardo et al. 2011a).

One major response to N deposition on terrestrial ecosystems is eutrophication or enrichment of an ecosystem with a limiting nutrient (Bobbink et al. 2010). Because plant growth in temperate terrestrial ecosystems tends to be primarily limited by N availability (Vitousek and Howarth 1991, Vitousek et al. 2002), increasing the inputs of this limiting nutrient often has a cascade of effects (Galloway et al. 2003). These include but are not limited to increased vascular plant production primarily aboveground (Stevens et al. 2015), decreased light at the soil level (Hautier et al. 2009), decreases in biodiversity and shifts in plant community composition (Clark and Tilman 2008, Hautier et al. 2009), enrichment of foliar concentrations of N (Bobbink et al. 2010), increased herbivory and pest damage (Throop and Lerdau 2004), direct losses of sensitive species such as lichen and bryophytes (Geiser et al. 2010, Root et al. 2015), and changes in the belowground populations of bacteria, mycorrhizal fungi, and non-mycorrhizal fungi (Lilleskov et al. 2002, 2008, Pardo et al. 2011a). Some of these processes can also be triggered by N (or S)-induced soil acidification, and separating these co-occurring stressors remains a challenge, although many are distinctly N-eutrophication effects. Each of these responses feedbacks and influences one another, and may aggregate to affect local and regional biogeo-chemical cycling as well as climate feedbacks (Arneth et al. 2010, Pinder et al. 2012). Terrestrial eutrophication effects are not restricted to natural ecosystems and processes alone, but also directly and indirectly affect human health and well-being, for example, through effects on human respiratory health, increased costs to drinking water, and impacts on recreation and ecosystems (Compton et al. 2011). The total damages from anthropogenic release of N in the United States are not trivial and have been estimated for the early 2000s to be $210 billion/yr (range: $81–441 billion/yr; Sobota et al. 2015).

Recent advances in two fields of study help us move toward a more precise quantification of the links between environmental degradation and human well-being. First, the maturation of research on “critical loads” of deposition for different ecosystems and ecological endpoints (Bobbink et al. 2010, Blett et al. 2014). A critical load is defined as “a quantitative estimate of an exposure to one or more pollutants below which significant harmful effects on specified sensitive elements of the environment do not occur according to present knowledge” (Nilsson and Grennfelt 1988). Over the past several years, critical loads for N, which used to be relatively understudied in the United States compared with Europe, have been developed for many environmental endpoints including impacts to lichen communities (Geiser et al. 2010, Stevens et al. 2012), herbaceous plant community composition (Fenn et al. 2010, Simkin et al. 2016), forest tree health (Thomas et al. 2010, Duarte et al. 2013), and for many other endpoints across the United States (Pardo et al. 2011a, b). Development of critical loads enables a quantitative link between N deposition and the risk to a specific ecological endpoint.

The second advancement is in the area of ecosystem services, to better link changes in a specific ecological effect with an ultimate human beneficiary. The term ecosystem services conveys the principle that natural systems provide socially and economically valuable goods and services deserving of protection, restoration, and enhancement (MEA 2005, Boyd and Banzhaf 2007). Ecosystem goods and services include the ecological features, qualities, or commodities society values, such as food, timber, clean drinking water, water available for irrigation, transportation, and industry, clean air, scenic beauty, and species important to us for recreational, ethical, or cultural reasons. Explicitly linking ecosystem services with affected people is difficult because of the broad definition of ecosystem services and the numerous types of services that could be affected. One strategy to address that challenge, and the focus of this paper, is to causally relate ecosystem stressors (in our case atmospheric deposition of N) to changes in Final Ecosystem Goods and Services (FEGS). Final Ecosystem Goods and Services are a subset of ecological outcomes, specifically the “components of nature, directly enjoyed, consumed, or used to yield human well-being” (Boyd and Banzhaf 2007). Final Ecosystem Goods and Services provide a bridge between ecological outcomes and analysis of their social costs and benefits, since by design they are the ecological outcomes most directly relevant to human use, enjoyment, and understanding. The U.S. EPA recently developed Final Ecosystem Goods and Services Classification System (FEGS-CS) to add structure and clarity to linking people with their local or regional environment (Landers and Nahlik 2013). Final Ecosystem Goods and Services connect specific human beneficiaries with ecological endpoints from environmental classes or types such as lakes, grasslands, and rivers. Previous classification systems did not attribute the user group for various services except in select clear cases (e.g., hunters and numbers of deer). The concept of linking FEGS to beneficiaries has been applied and refined by others (Bagstad et al. 2013, Ringold et al. 2013, Boyd et al. 2015, Wong et al. 2015). By making this link explicit between the effects of a stressor on a biological indicator and the user, or beneficiary, scientists can determine multiple possible links and select the biophysical metrics of most importance to different users. For example, a residential property owner may be negatively affected by terrestrial eutrophication through increased probability of fire, while a recreational photographer might be negatively affected if eutrophication is also associated with losses of native wildflower plant species.

These two advancements are integrated into the STressor–Ecological Production function–final ecosystem Services Framework (STEPS; Bell et al. 2017) to explicitly link human beneficiaries and end users to an initial shift in a biological indicator. We used this approach to examine the multitude of ecological impacts that occur with the exceedance of a single critical load, and to identify the FEGS most impacted by this environmental impact. The resulting model of how the ecosystem responds to critical load exceedance is an important step toward social and economic evaluation. To be clear, this paper does not conduct such social and economic evaluation. Rather, it is focused on the important, but more modest, task of identifying the biophysical linkages between loads and beneficiary-specific FEGS. The work sets the stage for subsequent monetary and non-monetary evaluation of load-driven FEGS changes. Here, we describe the outcome of a workshop to explicitly link exceedances of critical loads of terrestrial eutrophication to ecosystem services and human beneficiaries using this STEPS Framework. We acknowledge that eutrophication is not only a terrestrial issue, and that eutrophication and acidification often co-occur, and refer the reader to companion papers in this special issue that focus on aquatic eutrophication (Rhodes et al. 2017), and terrestrial acidification (Irvine et al. 2017), and aquatic acidification (O’Dea et al. 2017).

## Methods

Twenty-seven scientists and managers participated in a workshop organized by the National Park Service to establish relationships between air quality and impacts to FEGS (Blett et al. 2016). The participants covered a broad range of disciplines and experiences, including freshwater biologists, terrestrial ecologists, lichenologists, economists, social scientists, local park administrators, nonprofit researchers, and national air quality analysts that support the regulatory and conservation branches of government. The work-shop took place from 24 February 2015 to 26 February 2015, at Santa Monica Mountains National Recreation Area, in Thousand Oaks, California. The main goal of the workshop was to develop relationships between biological responses from critical load exceedances and FEGS, in four topical areas related to atmospheric deposition impacts: Terrestrial Eutrophication (this group), Terrestrial Acidification (Irvine et al. 2017), Aquatic Eutrophication (Rhodes et al. 2017), and Aquatic Acidification (O’Dea et al. 2017). Here, we describe the activities of the Terrestrial Eutrophication subgroup.

### STEPS Framework: Development of causal chains

**Fig. 1.**
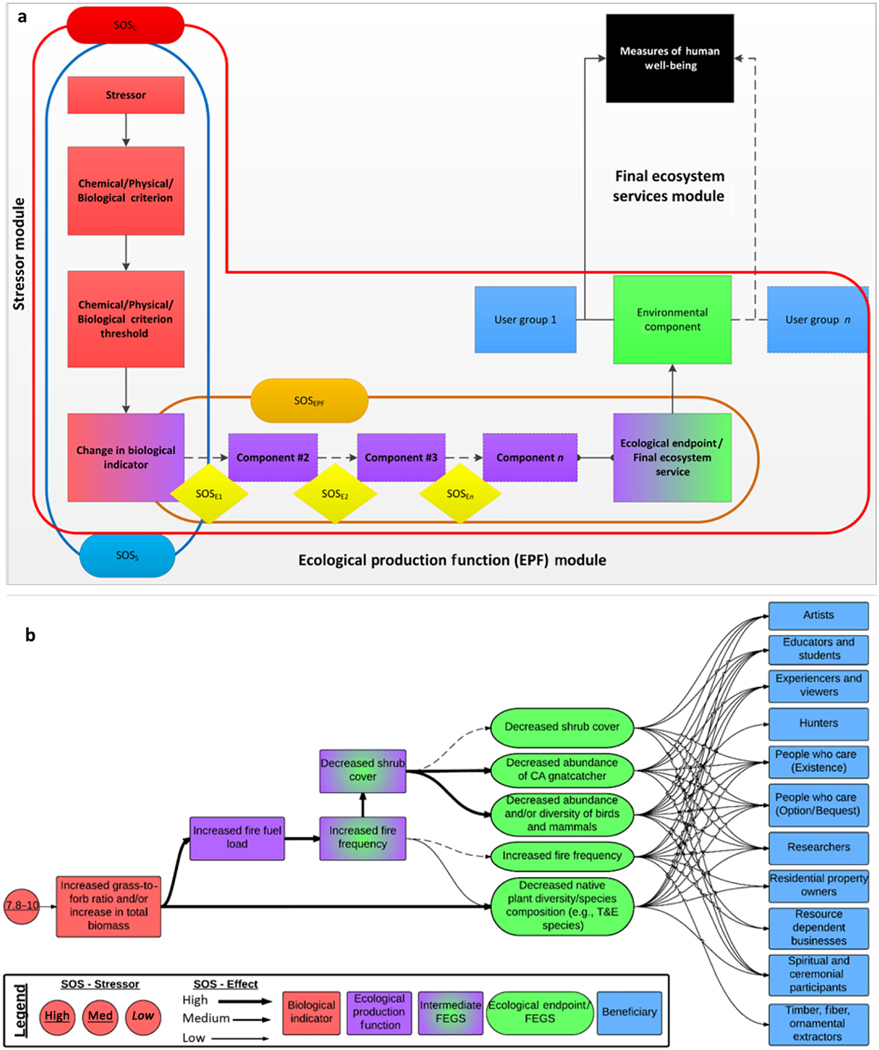
A conceptual model (a) of the STressor–Ecological Production function–final ecosystem Services Framework (Bell et al. 2017). The Stressor Module (red squares) consists of the chemical, environmental, and/or biological responses that are influenced by a stressor and lead to a change in the biological indicator. The Ecological Production Function (EPF) Module (purple squares) is the cascade of ecosystem effects due to the change in the biological indicator. The EPF can have zero to n additional steps which terminate at the ecological endpoint, synonymous with Final Ecosystem Goods and Services (FEGS). The ecological endpoint is the transition between the EPF and FEGS, and feeds into the Final Ecosystem Services Module which associates the endpoint with an Environmental Class/Subclass (e.g., forests, grasslands, lakes) and a beneficiary Class/Subclass. The SOS_S_ (blue line), SOS_EPF_ (orange line), and SOS_C_ (red line) represent the strength of science (SOS) of the relationships within the Stressor Module, EPF Module, or the entire chain, respectively. The yellow diamonds are the SOS_E_ score for individual steps in the EPF chain, which are used in the calculation of SOS_EPF_ (Eq. 1). Further details are available in Bell et al. (2017). In the example (b) from the coastal sage scrub of Mediterranean California, the critical load for changes in the biological indicator (i.e., increases grass-to-forb ratio and/or increase in total biomass) is 7.8–10 kg N·ha^−1^·yr^−1^ and is of medium SOS_S_ (Table 1). This directly affects one FEGS (i.e., decreased native plant diversity/species composition), and indirectly affects four others via an EPF that includes increased fire fuel loads, fire frequency, and sometimes also decreased shrub cover. The SOS for these individual effects varies from high to low (illustrated in the arrow sizes along the EPF only). These five FEGS ultimately affect 11 beneficiaries.

All topical areas used the STEPS Framework (Bell et al. 2017) to create a conceptual model of ecosystem responses to N (and for some groups S and/or P), to link the exceedance of a critical load to a FEGS and the associated human beneficiaries affected. The STEPS Framework consists of three modules: the Stressor, the Ecological Production Function (EPF), and the Final Ecosystem Services Modules (Fig. 1). The Stressor Module identifies how a change in environmental conditions affects a specific biological indicator. The EPF Module is the core of the STEPS Framework as it describes the series of cause and effect relationships that link the biological indicator of a stressor to an ecological endpoint that is directly used, appreciated, or valued by humans (i.e., a FEGS). An EPF is a chain of events by which ecosystems produce ecosystem services (Bell et al. 2017). We used the FEGS-CS in the final module of the STEPS Framework to classify the ecological endpoint as an ecosystem service by recognizing both its environmental element and the human groups who use or value the resource (i.e., beneficiary classes; Landers and Nahlik 2013). We use the term “FEGS” broadly within this paper to describe the ecological endpoint of an EPF and the term “beneficiary” to describe the human groups who use or value the resource.

The STEPS Framework is easily conceptualized with an example. In coastal sage scrub communities in the southwest, N deposition (Stressor) affects herbaceous community composition (Biological Indicator) by inducing a shift toward more invasive grasses and aboveground production (Change in Biological Indicator). In this example, the precursor “Chemical/Biological Criterion Threshold” might be a level of soil solution nitrate that induces a shift in composition, but it is not known for this chain and is skipped. Increases in invasive grasses and aboveground production lead to increases in fire fuel loads (Effect *i*), which ultimately can lead to increases in fire frequency and decreases in native species (both FEGS). Increases in fire frequency can affect homeowners among others (beneficiaries), and decreases in native species can affect recreational hikers among others.

We began by identifying known chemical and biological indicators for N deposition-induced terrestrial eutrophication. These indicators represent the first ecological response to N deposition that has a reported critical load. All critical loads were reported as a flux of N (kg ha^−1^ yr^−1^; e.g., Pardo et al. 2011a, b). We identified 21 known initial biological indicators with a published critical load for N deposition (Table 1). These represent a sub-set of all reported critical loads for eutrophication from the scientific literature (e.g., Pardo et al. 2011a, b). We did not attempt to develop chains for all known critical loads, but instead focused on particular areas based on expert judgment. From the subset selected, we developed EPFs linking these biological indicators to FEGS. We also did not attempt to describe all positive and negative effects on FEGS and beneficiaries because the intended focus was to understand what is *at risk* to terrestrial eutrophication (and critical loads by definition are for “harmful” effects), though we acknowledge that there are some FEGS that benefit from additional N up to some threshold (e.g., carbon sequestration, sometimes biodiversity at low N deposition rates). It is important to remember that a FEGS can exist within the EPF as well as at the end of the EPF, because different beneficiaries value different aspects of the environment. As an example, for mushroom collectors and mycologists, shifts in mycorrhizal fungi from N deposition may be a FEGS, but for a timber producer, shifts in fungal communities may not be a FEGS until it influences the aboveground production of trees.

It became clear that some biological indicators and EPFs were ecosystem-specific, so we differentiated chains by North American Level 1 Ecoregion (CEC 1997). When further geographic differentiation was appropriate and possible, ecoregions were divided into “ecosystems” identified in the source literature. Workshop participants developed EPFs for five Level 1 Ecoregions (Eastern Temperate Forests, Marine West Coast Forests, Mediterranean California, North American Deserts, and Northwestern Forested Mountains). Many of the EPFs identified are also relevant for the Great Plains Ecoregion, though we excluded the Great Plains from this effort because of time constraints and due to the pre-ponderance of agriculture and ranching. We divided ecoregions into ecosystems for Mediterranean California (four ecosystems: coastal sage scrub [CSS], grassland, mixed-conifer forest, and serpentine grasslands) and North American Deserts (three ecosystems: creosote bush shrubland, pinyon-juniper/Joshua tree woodland, sagebrush steppe; Table 1; Pardo et al. 2011a, b).

We then used the FEGS-CS system to identify the main beneficiaries of these FEGS (Appendix S1: Table S1). Beneficiaries were described by two categories from the FEGS-CS, the Class and the Sub-class. The beneficiary Subclass is the direct FEGS user (e.g., Hunters vs. Artists), while the beneficiary Class describes the broader category of use (e.g., Recreational vs. Inspirational, respectively). Thus, hereafter we focus on beneficiary Subclasses and use the term “beneficiary.” The set of relationships between the change in a biological indicator due to exceedance of a critical load to the beneficiary is called a “chain.”

### Assignment of strength of science

Following the development and organization of the causal chains using the STEPS Framework, we assigned a strength of science (SOS) to each link of each chain (Fig. 1). This was determined by the experts, using a three-level scale, according to the number of publications reporting that connection, and the agreement among those studies (Bell et al. 2017), similar to other synthesis efforts (e.g., Millennium Ecosystem Assessment; MEA 2005). There were two SOS scores given to relationships within the chains: (1) for the stressor (i.e., the critical load, SOS_S_) and (2) for the links between each component in the EPF (SOS_E_). These were used to calculate three diagnostic SOS scores for each chain (Fig. 1): (1) for the EPF (SOS_EPF_), (2) for the weakest link in the EPF (SOS_WL_), and (3) the SOS for the entire chain from the critical load to the FEGS (SOS_C_). Each SOS diagnostic score emphasizes a different attribute of the uncertainty associated with the chain. The SOS_S_ evaluates the SOS between the critical load and the initial biological indicator. The SOS_EPF_ 8, characterizes the EPF based on its length and the individual link scores (SOS_E_, via Eq. 1). The SOS_E_ scores are given values of high = 1, medium = 0.67, and low = 0.33. We assumed that the longer an EPF gets, the less confidence that confounding factors are not impacting the identified components. For this case study, the constant *M* is set to 8, suggesting that if an EPF is longer than six components, the potential complexity reduces the confidence in the relationships to zero.


(1) $${\text{SOS}}_{\text{EPF}}=\frac{\sum {\text{SOS}}_{\text{E}}}{\;\text{EPF}\;\text{Length}\;}\times \left(1-\frac{1}{M-\;\text{EPF}\;\text{Length}\;}\right).$$

The SOS_C_ represents the confidence across the entire chain, from the change in an indicator due to a stressor to the change in a final ecosystem service. Eq. 2 calculates the SOS_C_ by averaging the full weight of the SOS_S_ with the diminished value of each SOS_E_ based on the chain length. The SOS_S_ retains its full confidence because this is the basis of the analysis and the start of the measured change in the ecosystem. For those indicators that are also ecosystem services, the SOS_C_ score will be equal to the SOS_S_ value.


(2) $${\text{SOS}}_{\text{C}}=\frac{{\text{SOS}}_{\text{S}}+\left({\text{SOS}}_{\text{EPF}}\times \text{EPF}\;\text{Length}\right)}{\text{EPF}\;\text{Length}+1}.$$

The weakest link of the chains (SOS_WL_) was then determined by the lowest SOS score within the chain. This value allows for chains to be ranked based on the heuristic that a chain is only as strong as its weakest link.

## Results

### Overview of all chains examined

**Fig. 2.**
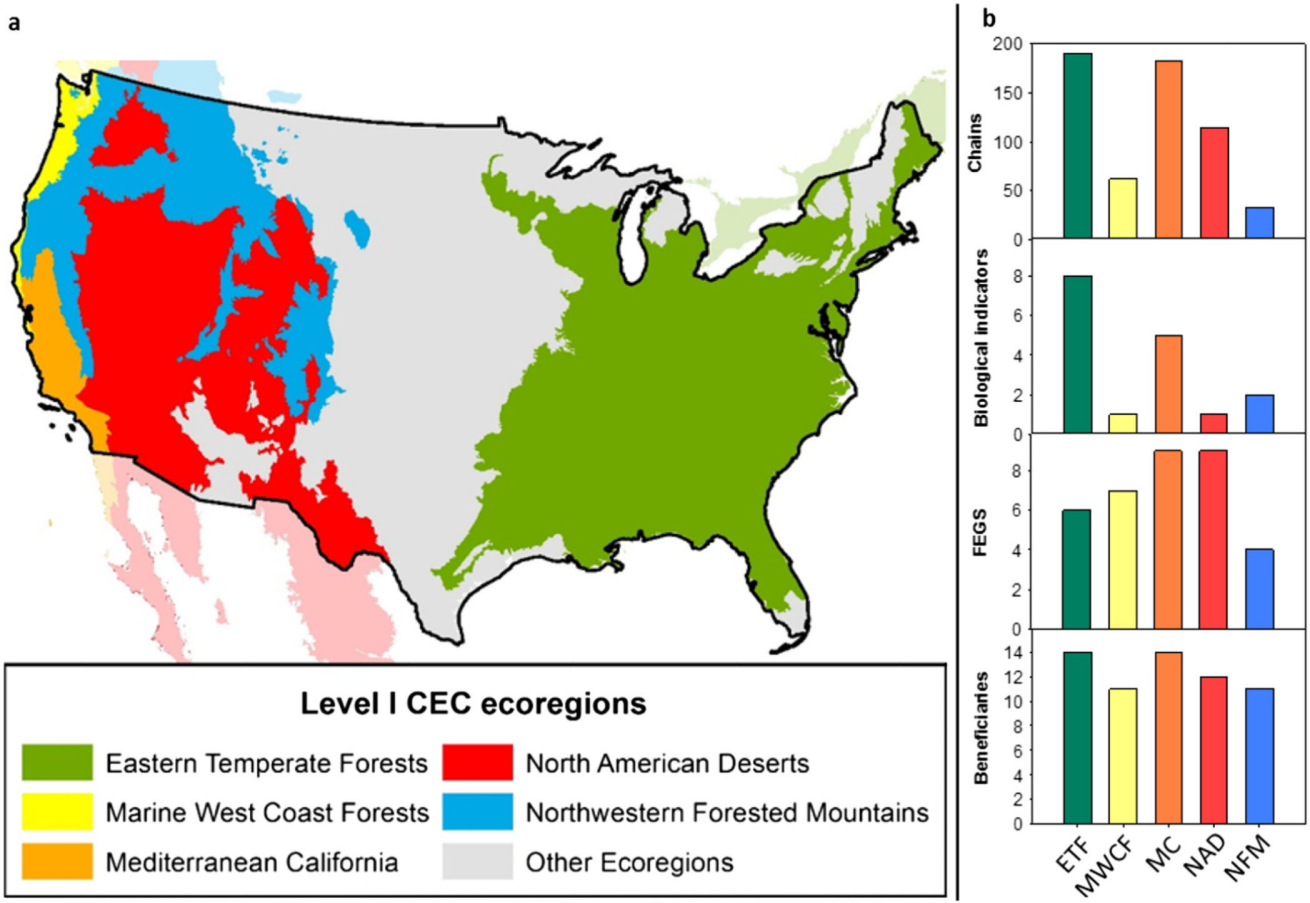
Map of Level 1 Ecoregions examined (a) and the count of unique chains, biological indicators, Final Ecosystem Goods and Services, and beneficiaries for each ecoregion (b).

Nitrogen deposition affected 21 system-specific critical loads related to terrestrial eutrophication, which cascaded through 582 chains (Table 1). This cascade was grouped into 76 EPFs that affected 66 total FEGS (21 unique) and 198 regional beneficiaries (17 unique) across all ecoregions (Table 1), some of which were affected in many ecoregions and ecosystems. Biological indicators were impacted by exceedances of various critical loads, ranging from 2.7 to 39 kg N·ha^−1^·yr^−1^, which differed widely by indicator and in some cases ecoregion (Table 1). Some biological indicators were present in some ecoregions and not others; and, the same biological indicator could be triggered at different critical loads in different ecoregions, and even ecosystems within an ecoregion. Some biological indicators were at the species level while others were at broader taxonomic groups. The number of unique biological indicators within an ecoregion was mainly driven by the number of distinct life forms represented in the literature. This led to the Marine West Coast Forests and North American Deserts each having only one biological indicator (lichen and grass: forb communities, respectively; Table 1), while the Eastern Temperate Forests and Mediterranean California had the most biological indicators and chains (Table 1, Fig. 2). Importantly, there were similar numbers of unique FEGS and beneficiaries affected across ecoregions (Fig. 2), even though the total numbers could be quite different depending on the number of biological indicators (Table 1).

### Patterns among ecoregions

**Fig. 3.**
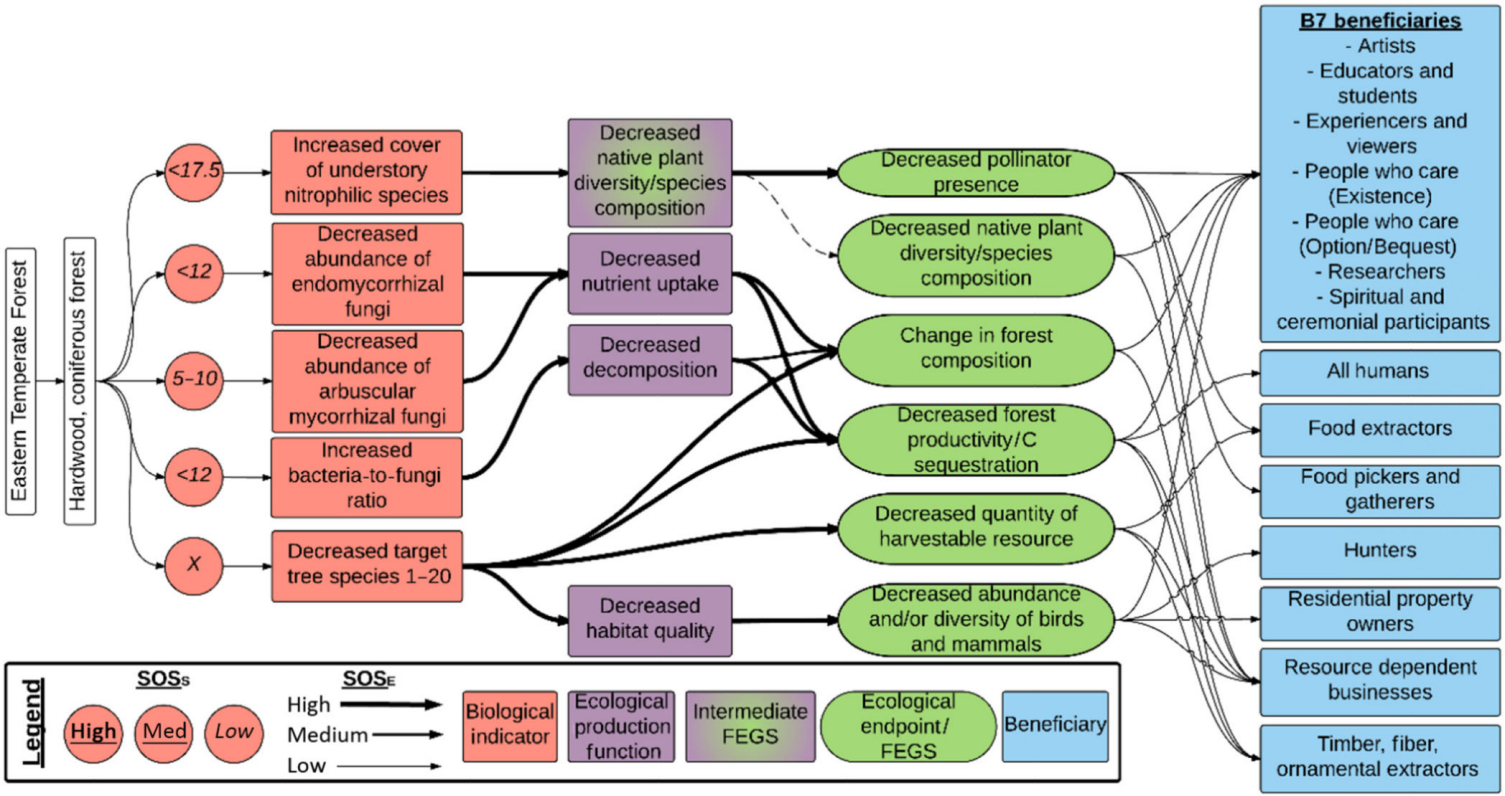
Diagram of the chains assessed for terrestrial eutrophication impacts in the Eastern Temperate Forests Ecoregion. Colors and symbols are as in Fig. 1, and dashed lines are used to identify intermediate Final Ecosystem Goods and Services (FEGS) in the Ecological Production Function that are also final FEGS.

**Fig. 4.**
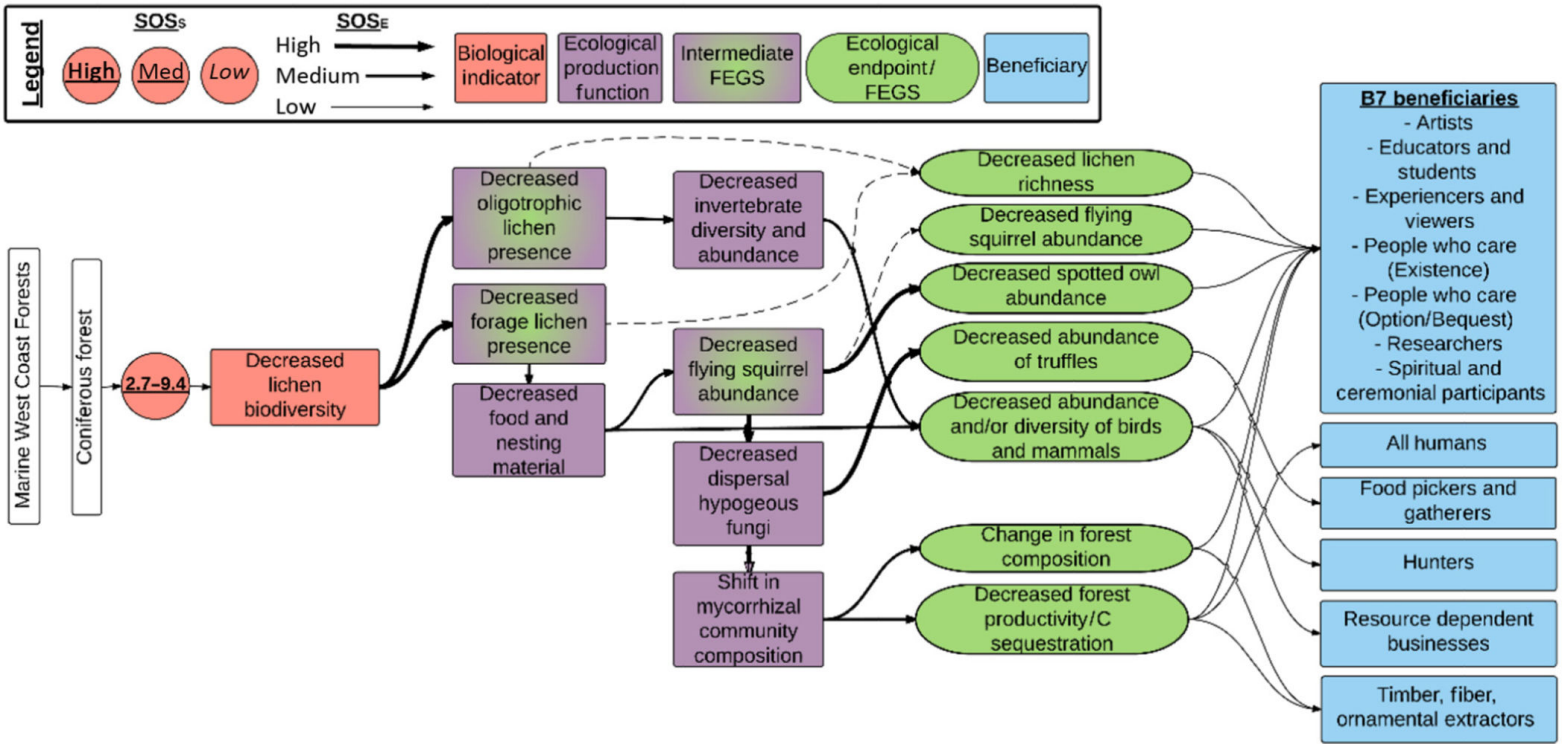
Diagram of the chains assessed for terrestrial eutrophication impacts in the Marine West Coast Forests Ecoregion. Colors and symbols are as in Fig. 1, and dashed lines are used to identify intermediate Final Ecosystem Goods and Services (FEGS) in the Ecological Production Function that are also final FEGS.

**Fig. 5.**
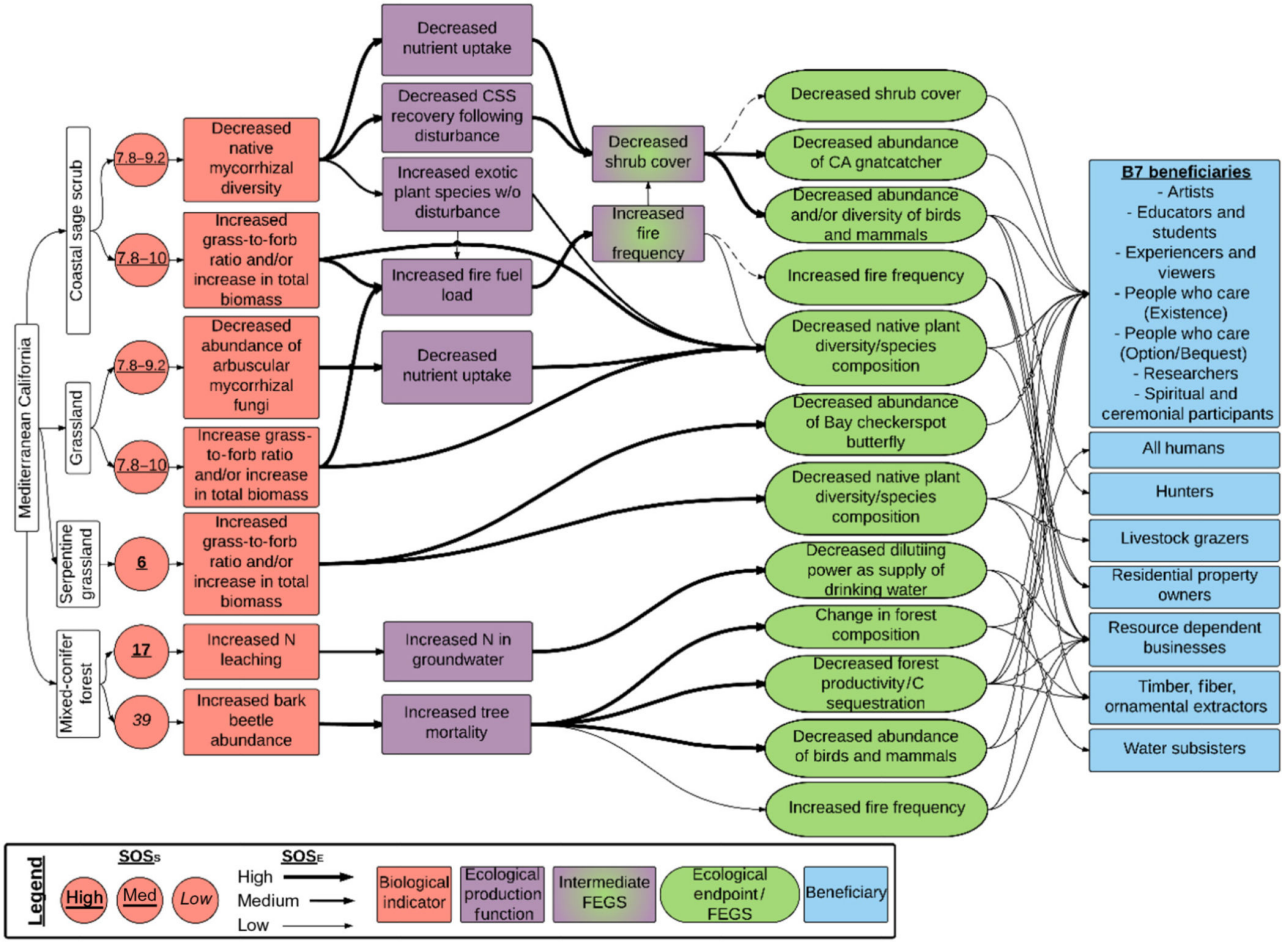
Diagram of the chains assessed for terrestrial eutrophication impacts in the Mediterranean California Ecoregion. Colors and symbols are as in Fig. 1, and dashed lines are used to identify intermediate Final Ecosystem Goods and Services (FEGS) in the Ecological Production Function that are also final FEGS.

**Fig. 6.**
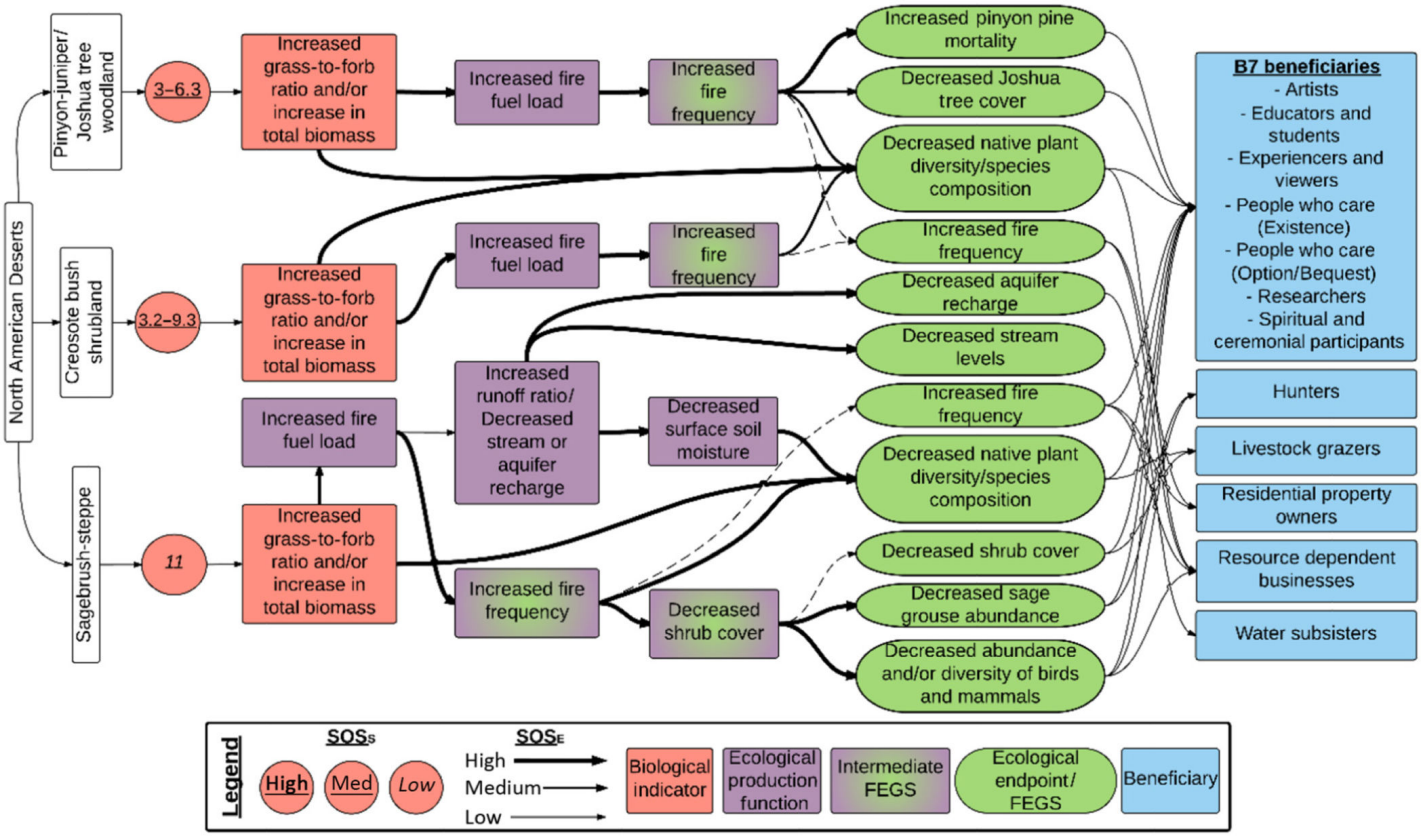
Diagram of the chains assessed for terrestrial eutrophication impacts in the North American Deserts Ecoregion. Colors and symbols are as in Fig. 1, and dashed lines are used to identify intermediate Final Ecosystem Goods and Services (FEGS) in the Ecological Production Function that are also final FEGS.

**Fig. 7.**
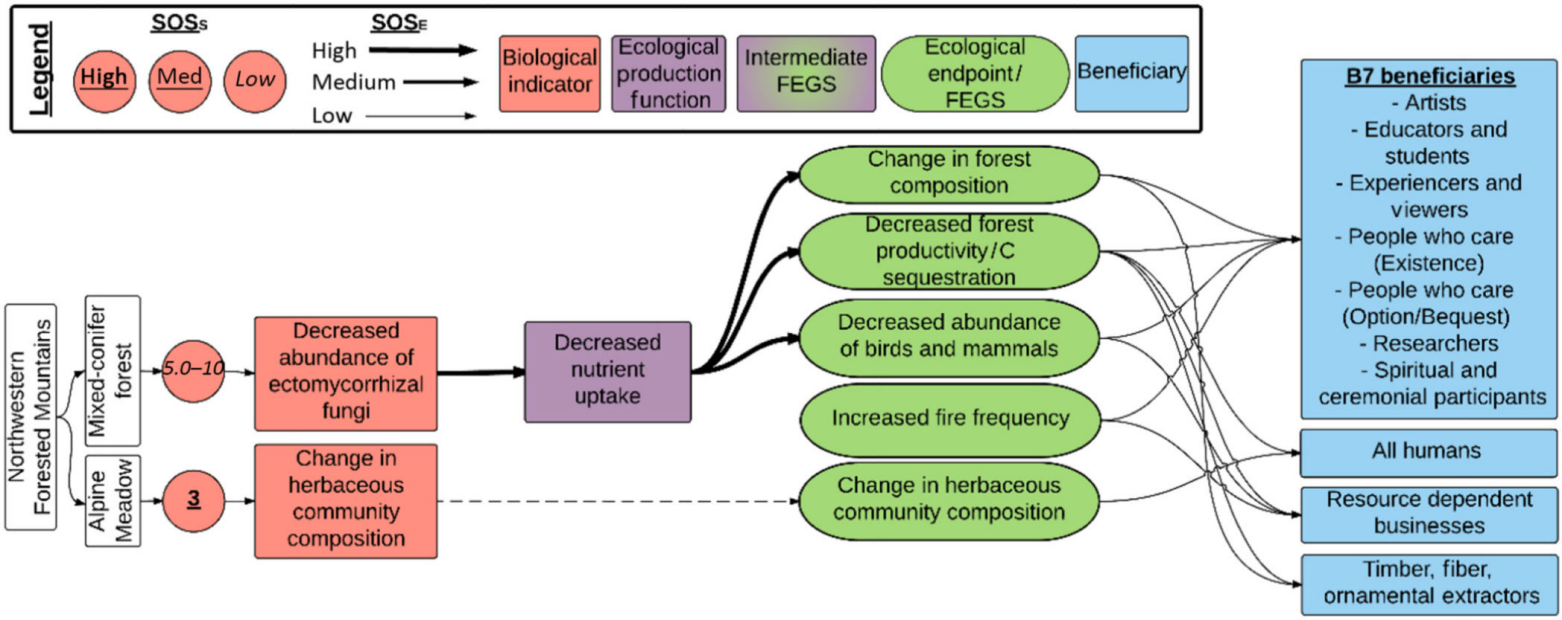
Diagram of the chains assessed for terrestrial eutrophication impacts in the Northwestern Forested Mountains Ecoregion. Colors and symbols are as in Fig. 1, and dashed lines are used to identify intermediate Final Ecosystem Goods and Services (FEGS) in the Ecological Production Function that are also final FEGS.

Most terrestrial eutrophication chains were initiated by changes in biological indicators associated with grasses and/or forbs (192 chains, 34% of all chains), mycorrhizal communities (129 total, 22%), tree species (121 total, 21%), and lichen biodiversity (62, 11%; Table 2). Together, these accounted for almost 90% of chains assessed. Differences in numbers of chains associated with different biological indicators likely reflect greater research for some taxonomic groups rather than greater ecological importance for these indicators. A visual representation of the cascading effects of the change in biological indicators is presented in Figs. 3–7. Each biological indicator affected an average of 3.9 FEGS and 9.9 beneficiaries. There was large variation in the number of FEGS affected by each biological indicator, while the number of beneficiaries was more similar among biological indicators (Table 2).

The 21 unique FEGS identified (Table 3) covered a wide range of effects, including biogeo-chemical responses (e.g., carbon sequestration, N cycling), population responses (e.g., flying squirrel abundance), as well as hydrologic responses (e.g., decreased aquifer recharge), among others. Water quality effects were addressed in the Aquatic Eutrophication group (Rhodes et al. 2017). Some of the 21 unique FEGS are considered both final and intermediate ecosystem services, depending on the beneficiary. Nearly 75% of chains were associated with the five most common FEGS, mostly associated with changes in plant and animal communities, decreased forest productivity and carbon sequestration, and increased fire frequency (Table 3; see Appendix S1: Table S1 for linkages between each FEGS and the corresponding beneficiary(ies)). The remaining 16 FEGS affected fewer chains; however, this asymmetry again may reflect the amount of research in an area as opposed to a magnitude of effect.

The 66 FEGS (21 unique) were utilized by 198 beneficiaries (17 unique), with each FEGS affecting an average of 6.7 beneficiaries (Table 3). Seventeen FEGS affected 7–10 beneficiaries each, with the remaining four FEGS affecting one to two beneficiaries (Table 3). Common among these 17 FEGS was a set of seven beneficiaries (hereafter termed “B7 beneficiaries”) that appeared together: Artists; Educators and Students; Experiencers and Viewers; People Who Care (Existence); People Who Care (Option/Bequest); Researchers; Spiritual and Ceremonial Participants and Participants of Celebration. The B7 beneficiaries were affected by most chains, present across all ecoregions, and almost always together (Tables 3, 4).

### Patterns within ecoregions

#### Eastern Temperate Forests.—

**Fig. 8.**
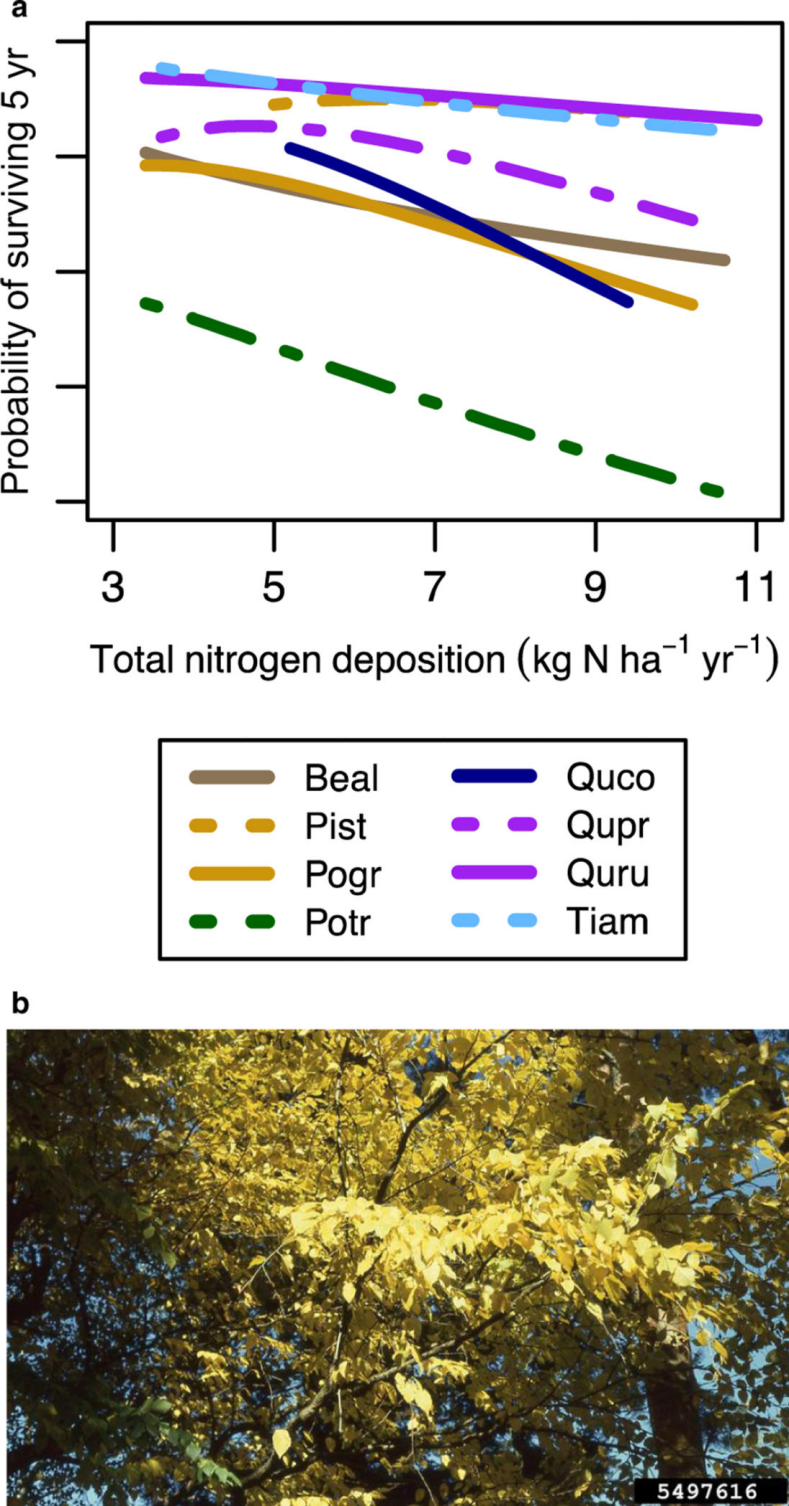
Survival (a) of eight tree species in Thomas et al. (2010) were found to decrease with N addition (Beal: Yellow birch, Pist: White pine, Pogr: bigtooth aspen, Potr: trembling aspen, Quco: scarlet oak, Qupr: chestnut oak, Quru: red oak, Tiam: basswood). A photograph (b) of trembling aspen that has been identified as sensitive to N-induced eutrophication (T. Davis Sydnor, The Ohio State University, Bugwood.org).

The Eastern Temperate Forests Ecoregion had the most biological indicators (8) and chains (190) represented in the database, with a range of critical loads from <4 to <17.5 kg ha^−1^ yr^−1^ (Table 1). The SOS for the critical load (SOS_S_) was generally low except for critical loads from Thomas et al. (2010) analyzing tree species growth and mortality responses across a multi-state area in the east. Biological indicators included three related to changes in soil biota (decreased abundance of arbuscular mycorrhizal fungi, decreased abundance of ectomycorrhizal fungi, increased bacteria-to-fungi ratio), one related to understory herbs (increased cover of understory nitrophilic species), and four associated with specific tree species. We only developed four tree species-specific chains from the set of 24 species assessed in Thomas et al. (2010), because these four species were the strongest (>5%) negative responders for growth (red pine [*Pinus resinosa*]) or survival (bigtooth aspen [*Populus grandidentata*], scarlet oak [*Quercus coccinea*], and trembling aspen [*Populus tremuloides*]). Of the remaining 20 species, only seven responded negatively but with a weaker (<5%) magnitude of effect (Thomas et al. 2010). Thus, we focus on these four species as the most sensitive in this ecoregion based on our current understanding. Sugar maple has also been found to be sensitive to atmospheric deposition on this region (Sullivan et al. 2013), although it appears this is more an acidification effect than a eutrophication effect, as other studies have found weaker (Duarte et al. 2013) or positive (Thomas et al. 2010) responses for sugar maple. Perturbation of these eight biological indicators was linked to changes in forest composition and/or function, which ultimately affected six FEGS (Table 1, Figs. 2, 3). These six FEGS broadly were associated with changes in forest community structure (change in forest tree composition, decreased abundance of birds and mammals, decreased pollinator presence, decreased native plant diversity), forest function (decreased forest productivity/C sequestration), and changes in forest products (decreased quantity of harvestable resource, decreased quality of fall foliage). Final Ecosystem Goods and Services, such as timber, maple syrup, or other extractable resources, are lumped together with a decreased quantity of harvestable resources in order to separate the extractable (e.g., timber) and non-extractable (e.g., views of fall foliage) resources. There were 13 beneficiaries affected, including the B7 beneficiaries set plus All Humans, Food Extractors, Food Pickers and Gatherers, Hunters, Resource-Dependent Businesses, and Timber, Fiber, and Ornamental Extractors. Detailed linkages between each FEGS and the corresponding beneficiary(ies) are provided in the Data S1. As an example, decreased growth of red pine was associated with the FEGS of change in forest composition, which ultimately affected eight beneficiaries including the B7 beneficiaries along with Timber, Fiber, and Ornamental Extractors. Splitting out the B7 was redundant, but to illustrate, reduced growth of red pine could negatively impact artists who may paint them, students and educators who may study them, experiencers and viewers who may explore the area, people who care about their existence, people who would like the option to explore the area and/or bequeath that option to their children, researchers who may study them, and local indigenous cultures who may use red pine in their ceremonies. For the spiritual/cultural uses, the Algonquin, Ojibwa, Potawami, and Chippewa Tribes used the bark, cones, and leaves for medicinal purposes, to relieve headache, treat a cold, and revive a comatose patient (NAEB 2016). Almost all tree species examined had a spiritual/cultural use, and almost all FEGS were enjoyed by the B7 beneficiaries as a set. For the Timber, Fiber, and Ornamental Extractors, red pine are known to be used for lumber, pilings, poles, cabin logs, railway ties, posts, mine timbers, box boards, pulpwood, and fuel, and are also planted as an ornamental (Hauser 2008). Thus, for simplicity we focus subsequently on groups of beneficiaries that are affected by groups of FEGS, rather than individual connections that are presented in Data S1. The only beneficiaries not affected by FEGS from the Eastern Temperate Forests were Water Subsisters, Livestock Grazers, and Residential Property Owners, the latter two of which could probably have been included but were not considered major beneficiaries by the workshop participants (Blett et al. 2016). Box 1 describes some of the various FEGS and beneficiaries impacted by changes in the Eastern Temperate Forests Ecoregion in more detail (see also Fig. 8).

#### Marine West Coast Forests.—

**Fig. 9.**
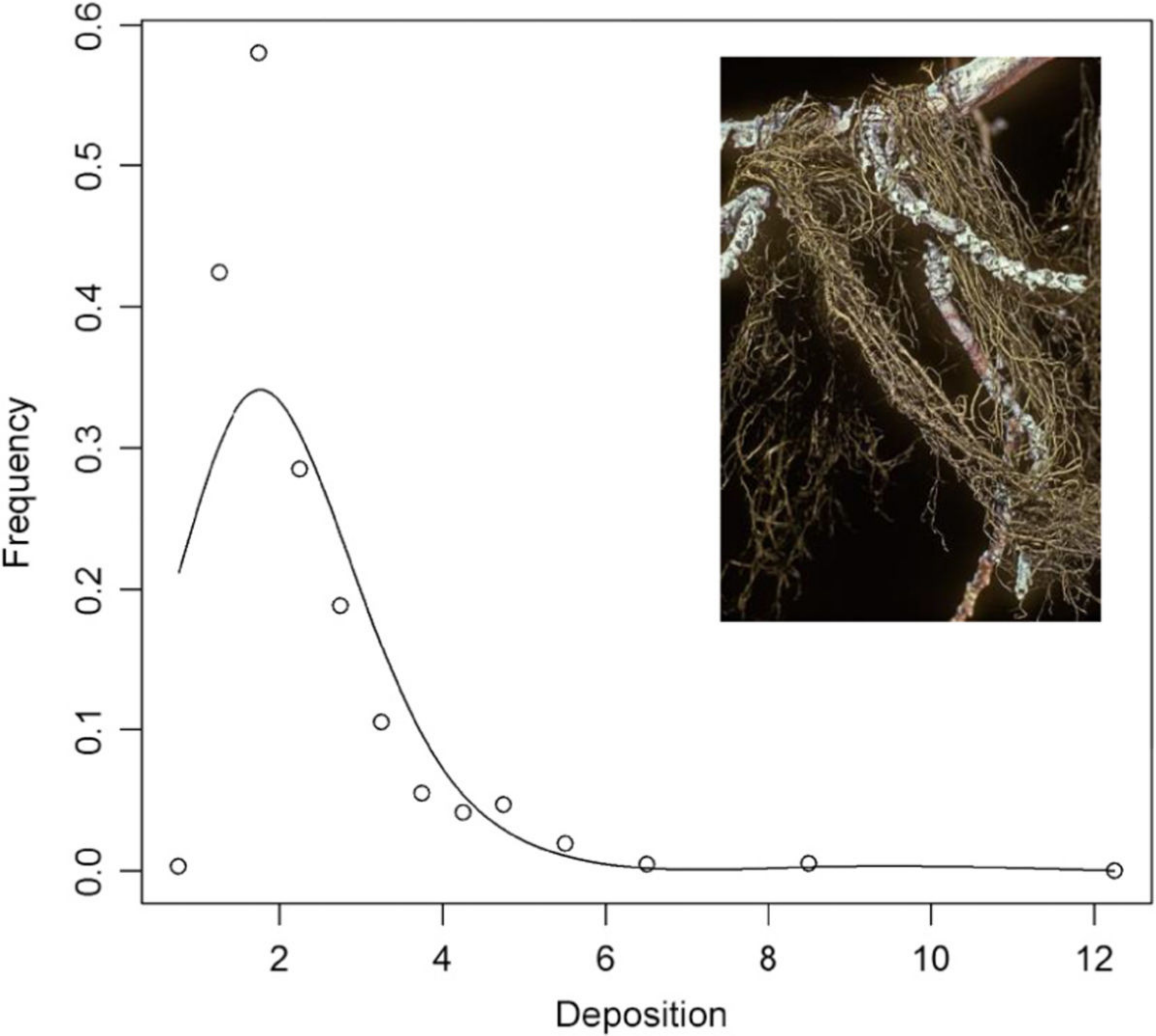
Frequency of occurrence of horsehair lichen (*Bryoria fremontii*) across a range of nitrogen deposition (kg·ha^−1^·yr^−1^, USFS 2016) along with a photograph (insert). Photograph courtesy of Steven Sharnoff.

The Marine West Coast Forests Ecoregion had only one biological indicator reported, decreases in lichen biodiversity, which influenced 62 chains (Table 1, Figs. 2, 4). The critical loads varied from 2.7 to 9.2 kg ha^−1^ yr^−1^ and differed by ecosystem and threshold used to assess sensitivity (Geiser et al. 2010). We considered these very robust critical loads estimates and scored the SOS_S_ as high. Decreased lichen biodiversity occurred via either decreased forage lichen and/or decreased oligotrophic lichen, both of which affect many forest insect, bird, and mammal populations (Fig. 2). Ultimately, changes in this biological indicator either directly or indirectly affected seven FEGS (Fig. 4). These seven FEGS embodied many forest attributes, including plant, lichen, mammal, bird, and insect populations (see Box 2 and Fig. 9). Together, these FEGS were utilized by 11 beneficiaries, including the B7 beneficiaries plus All Humans, Food Pickers and Gatherers; Hunters; Resource-Dependent Businesses; and Timber, Fiber, and Ornamental Extractors (Data S1). See Box 2 for an example of how adverse effects on lichens can affect the structure and function of forests.

#### Mediterranean California.—

**Fig. 10.**
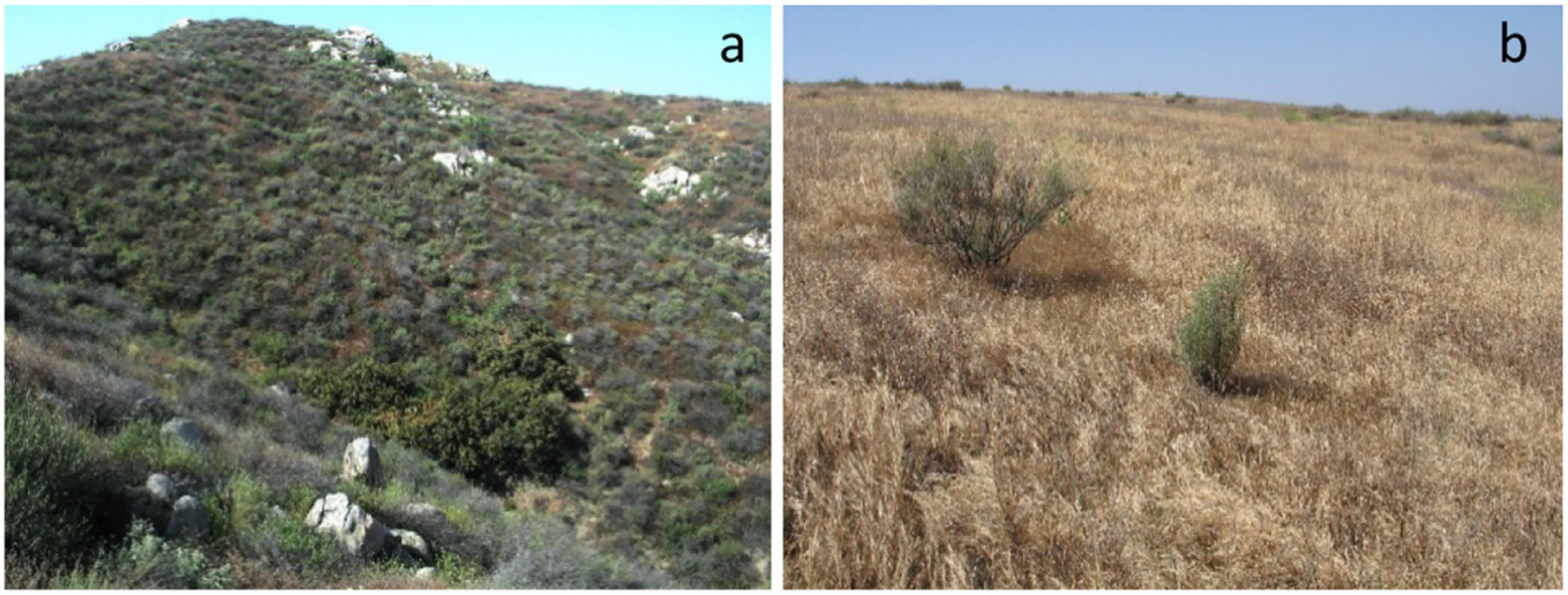
The mosaic structure of the coastal sage scrub community (a) can break down and give rise to exotic grasslands if the area is subjected to high fire frequency (b). Photographs courtesy of Robert J. Steers.

The Mediterranean California Ecoregion is an extensively studied system that was subdivided into four ecosystems: CSS, grassland, mixed-conifer forest, and serpentine grasslands (Fig. 5). Critical loads varied from 6 to 39 kg ha^−1^ yr^−1^, and the SOS_S_ ranged from low to high (Table 1). The majority of chains were for the CSS Ecosystem (99 of 183; Table 1), followed by mixed-conifer forests (36), grasslands (32), and serpentine grasslands (16). Three of the four ecosystems were affected by increased grass:forb ratio and/or increase in total biomass (CSS, grassland, serpentine grassland) with the fourth having distinct biological indicators related to leaching and pests (mixed-conifer forest; Fig. 2). Chains for the CSS were triggered by increasing dominance of grasses (grass:forb) and increases in total biomass and decreases in native mycorrhizal diversity. These changes led to several intermediate effects, including increased fire fuel load, decreased P and water uptake, and decreased shrub cover. Ultimately, these shifts affected five FEGS enjoyed by 10 beneficiaries (Table 1). The affected FEGS included decreased abundance of protected species (California gnatcatcher), increased fire frequencies, decreased abundance of birds and mammals, and changes in plant community composition. Beneficiaries included the B7 beneficiaries plus All Humans, Hunters, Livestock Grazers, Residential Property Owners, Resource-Dependent Businesses, Water Subsisters, and Timber, Fiber, and Ornamental Extractors. There were fewer affected FEGS in the grassland and serpentine grassland Ecosystems (Table 1), though a similar number and types of beneficiaries. The mixed-conifer forest had unique chains reported for this ecoregion, with increasing bark beetle abundance affecting forest structure, function, and fire regime; and increasing N leaching to the groundwater affecting aquifer resources (Fig. 2). Ultimately, these five FEGS in the mixed-conifer forest affected 13 beneficiaries that were similar for the other ecosystems (Data S1). See Box 3 and Fig. 10 for more details on how changes in the CSS Ecosystem are affected by terrestrial eutrophication.

#### North American Deserts.—

The North American Deserts Ecoregion has also been extensively studied and was subdivided into three ecosystems: sagebrush steppe, pinyon-juniper woodland, and creosote bush shrubland (Table 1, Fig. 6). All three had the same initial biological indicator, increased in grass:forb and/or increase in total biomass, though, with critical loads that ranged from 3 to 11 kg ha^−1^ yr^−1^ and varied in SOS_S_ from moderate to low across ecosystems. The most chains affected were in the SS (60) which had the lowest SOS_S_, but still a substantial number of chains were affected in the creosote bush shrublands (31) and the pinyon-juniper woodlands (23). While the indicator type in each ecosystem is the same, each of these areas is impacted by a different dominant invasive grass: *Schismus barbatus* in creosote bush shrubland, *Bromus rubens* in pinyon-juniper wood-lands, and *Bromus tectorum* in sagebrush steppe (Chambers et al. 2007, Rao and Allen 2010). All chains across ecosystems included increased fire fuel loads and fire frequencies, ultimately affecting nine FEGS for the ecoregion related to the plant community (decreased Joshua tree cover, decreased native plant species, decreased shrub cover, increased pine mortality), animal populations (decreased abundance of birds and mammals, decrease in game animals [e.g., sage grouse]), hydrology and water quality (decreased aquifer recharge, decreased stream levels), and disturbance (increased fire frequency). Twelve beneficiaries were affected, including the B7 beneficiaries, plus Hunters, Livestock Grazers, Residential Property Owners, Resource-Dependent Businesses, and Water Subsisters (see Data S1 for details).

#### Northwestern Forested Mountains.—

The North-western Forested Mountains Ecoregion was sub-divided into two ecosystems: alpine meadows and mixed-conifer forests (Table 1, Fig. 7). Each had a single biological indicator, with low critical loads with high confidence (alpine meadow, 3 kg ha^−1^ yr^−1^), or higher critical loads with lower confidence, expressed as a range (mixed-conifer forests, 5–10 kg N ha^−1^ yr^−1^; Table 1). Changes in herbaceous community composition occurred in the alpine meadow ecosystem, affecting one FEGS (Changes in herbaceous community composition) and eight beneficiaries (the B7 beneficiaries plus All Humans). This FEGS was not combined with the other herbaceous FEGS (e.g., increased grass:forb and increased biomass), because response to N deposition is different in this system, and is associated with an increase in cover of a sedge species (*Carex rupestris*) with no change in species richness or diversity (Bowman et al. 2006, 2012). The mixed-conifer forest responds with a decrease in ectomycorrhizal fungi, affecting three FEGS related to changes in forest composition, function, and diversity of birds and mammals (Fig. 7). Eleven beneficiaries were affected in this ecosystem including the B7 beneficiaries plus All humans, Hunters, Resource-Dependent Businesses, and Timber, Fiber, and Ornamental Extractors (Data S1).

### Strength of science

Broad differences in the SOS at the ecoregion level emerged (Table 5). The SOS_S_ was highest in the Marine West Coast Forests (1.0), and lowest in the North American Deserts (0.47) and North-western Forested Mountains (0.46) and intermediate in Eastern Temperate Forests and Mediterranean California Ecoregions (0.71–0.77). The high SOS_S_ in the Marine West Coast Forests occurred because all critical loads were based on decreases in lichen biodiversity and were scored as high (1.0, Table 1). The SOS_EPF_ was more similar across ecoregions (range: 0.67–0.83). Due to the length of the chains, the Marine West Coast Forests had the lowest average SOS_EPF_ score. The SOS_WL_ had similar rankings among ecoregions as SOS_S_, which were lowest for Northwestern Forested Mountains and North American Deserts. Within an ecoregion, at least one EPF had all SOS_E_ scores ranked as high, but due to the number of components, the high scores ranged from 0.83 to 0.86. The minimum scores ranged from 0.43 (Marine West Coast Forests) to 0.83 (Northwest Forested Mountains; Table 5). Only one chain had a SOS_S_ score of 1.0, indicating that the change in biological indicator was valued as a FEGS.

## Discussion

### Overview

We found that terrestrial eutrophication affected all ecoregions examined. The most chains were reported for the Eastern Temperate Forests, Mediterranean California, and North American Deserts (all > 100), and the fewest chains in the Northwestern Forested Mountains (N = 33). Even though there were different numbers of chains, we found similar numbers of unique beneficiaries affected among ecoregions. The distribution of chains, FEGS, and beneficiaries among ecoregions may be more a product of the level of research on terrestrial eutrophication in each ecoregion rather than the level of effect of terrestrial eutrophication. Nonetheless, these results represent our current state of knowledge and suggest that impacts from terrestrial eutrophication are not restricted to high deposition areas (e.g., Eastern Temperate Forests Ecoregion, portions of the Mediterranean California Ecoregion), but rather are widespread across the continental United States.

There were differences in the number and identity of biological indicators among ecoregion. Differences in number are expected, with low deposition areas reporting effects on fewer biological indicators that are often more sensitive. For example, only one biological indicator was reported in both the Marine West Coast Forests (decreased lichen biodiversity) and the North American Deserts (increased grass:forb and/or total biomass). These are generally low deposition ecoregions, and thus, only the most sensitive endpoints are reported as affected.

On the other end of the spectrum was the Eastern Temperate Forests Ecoregion, with eight bio-logical indicators (Table 1). This ecoregion has experienced much higher historical deposition rates, and thus, more numerous and varied indicators are reported as affected, including soil biota, understory herbs, and overstory trees. Even so, eight indicators is likely a lower bound for this ecoregion given that (1) Thomas et al. (2010) only examined a subset of 24 common tree species in the Northeastern United States (and only four of which responded with >5% reduction in growth or survival), and (2) we did not include lichen, a known sensitive taxonomic group, in our assessment of this ecoregion. As more tree species are examined, we may discover that more are vulnerable to terrestrial eutrophication. We did not include lichen as a biological indicator in the Eastern Temperate Forests because the published critical load (Pardo et al. 2011a, b) had the lowest reliability, based on expert judgment and extrapolation from the Marine West Coast Forests (Geiser et al. 2010). This sensitive life form is likely also impacted in the east, though it is more difficult to ascertain because of the lack of low deposition reference sites. Nonetheless, as more information becomes available it is likely that we will discover that more endpoints are affected in this ecoregion. Indeed, a recent study reported exceedances for herbaceous biodiversity across much of the east (Simkin et al. 2016) and found that critical loads were much lower than previously reported. This information, however, was not available at the time of the workshop and so is not included here.

The focus of research in the east has generally been on acidification as opposed to eutrophication, because of the high historical sulfur and acid deposition. As acid deposition has declined from its peaks in the 1970s and 1980s, forested systems are beginning to show signs of recovery from acidification (Lawrence et al. 2015). Whether recovery from eutrophication will occur simultaneously, lagged, or whether recovery from acidification will make eutrophication effects more apparent, remains unknown. Roof studies from the NITREX-EXMAN experiments in Europe in the 1990s that reduced incident N and S deposition demonstrated that some “fast cycling” processes can recover within a few years (e.g., nitrate leaching, foliar N), while other “slow cycling” processes may not (e.g., decomposition, vegetation composition; Boxman et al. 1998, Gundersen et al. 1998). Furthermore, even as deposition levels have stabilized or decreased in the eastern United States over the past decade, riverine nitrate is still increasing in some catchments and decreasing in others (Argerich et al. 2013). In the east, stream total nitrogen concentrations appear to be decreasing roughly north of Virginia between 2002 and 2012, and increasing south of Virginia (Oelsner et al. 2017), even though deposition has generally declined over both regions over this period. This suggests regional or even local factors at play. Indeed, Argerich et al. (2013) even found variation in the direction of change within a watershed. This suggests that recovery of terrestrial ecosystems does not directly follow decreases in deposition, and may include complex regional and local feedbacks preventing or delaying recovery. Other studies have also shown that reduction in soil N levels, once elevated, can require significant intervention to induce recovery of biogeo-chemical processes and biotic communities (Bakker and Berendse 1999, Clark and Tilman 2010, Jones et al. 2016). Furthermore, once eutrophication and changes in plant community composition have occurred, positive feedbacks maintaining the new community can further limit recovery, including sustained high N mineralization rates and recruitment limitation from individuals that are no longer present in the regional pool (Clark and Tilman 2010, Isbell et al. 2013). Recovery appears more rapid if soil amendments are made to restore favorable soil conditions and if local propagules are available (Clark and Tilman 2010, Storkey et al. 2015).

The focus on research in the west has generally been more on eutrophication as opposed to acidification, because of the generally lower deposition rates and often more alkaline soils. The observation that changes in herbaceous species was reported as a biological indicator across nearly all western ecoregions is driven by at least two factors. First, herbaceous species are reported to be more sensitive than trees to terrestrial eutrophication because of their high relative growth rates, shallow root systems, and shorter life histories (Pardo et al. 2011a). Thus, in lower deposition areas in the west lichen communities and herbs are the taxonomic groups being studied for eutrophication effects. Second, there is a long and rich history of research on herbaceous communities, nutrient limitation, and N enrichment, with some of the seminal works originating from these systems especially in high deposition areas of the west (e.g., Weiss 1999). Thus, as greater research accumulates for other taxonomic groups this emphasis in the west may or may not diminish. The ubiquity of N limitation in terrestrial ecosystems (LeBauer and Treseder 2008) and in particular for many western forests (Chappell et al. 1991) suggests that tree growth in the west may also be affected by N deposition. This effect might lead to a small net increase in aboveground tree biomass especially in well-buffered soils not vulnerable to acidification, which could be a net positive to some beneficiaries (e.g., timber producers), and a net negative to others if it is also associated with changes in the extant forest community composition (e.g., the B7 beneficiary Group).

This asymmetry of research emphasis in the east vs. west likely also explains some of the idiosyncrasies among indicators and FEGS among ecoregions. For example, the fact that shifts in lichen composition along deposition gradients were not included in the east is likely a result of much more emphasis on lichen research in the west than the east, rather than a lack of an effect in the east. For example, the Northern Parula (*Setophaga americana*) is an eastern warbler known to be dependent on the Ursula lichen for nesting, and flying squirrels are not restricted to the west. In addition, the observation that tree species responses are not emphasized in the west could be because of the prevailing view that trees are less sensitive to low N inputs (Pardo et al. 2011a, b), or that forests are more prone to acidification than eutrophication, even though recent empirical findings suggest that growth and mortality for some tree species are affected by low N deposition <5 kg·ha^−1^·yr^−1^ (Thomas et al. 2010). The mechanism was not empirically examined by Thomas et al. (2010) and remains an active area of research, but appears to be related to the mycorrhizal association of the tree species. All five tree species that were associated with arbuscular mycorrhizae, whose symbionts are unable to aid in the breakdown of soil organic N, tended to respond positively. All three species with negative growth responses were evergreen conifers with ectomycorrhizal associations. However, there were exceptions on the negative responses, with many evergreen conifers with ectomycorrhizal associations that did not respond to N at all or responded positively, and non-conifers with ectomycorrhizal associations that also did not respond to N at all or responded positively (Thomas et al. 2010). Thus, it appears to be a combination of traits that confers some vulnerability to N deposition. These two examples (emphasis on lichen in the west and trees in the east) also underscore the limitations of carrying out this investigation in a three-day workshop with a limited but representative scientists participating. This effort is intended to be a representative sample of the biological indicators, ecosystem services, and beneficiaries impacted, not an exhaustive review of these.

Although the identity of FEGS differed across ecoregions and ecosystems, the basic architecture of effect was similar—species of interest are affected or lost, community composition changes, and disturbance may also increase. The exact sequence of responses can take many forms and impact a regionalized subset of beneficiaries with different levels of intensity. Lumping FEGS into large categories (e.g., changes in plant or animal communities) more clearly illustrates the shared interests in avoiding terrestrial eutrophication across the United States. We broke down specific responses in some of the ecoregions (e.g., California gnatcatcher habitat in the west, fall foliage in the east, etc.) as examples of how specific, “high value,” resources change due to eutrophication. These are associated with fewer chains than the general FEGS above because they are more localized, species-specific responses. Disaggregating more of the responses reported here into species-level responses, and linking these to more specific beneficiary groups is a promising next step. See Irvine et al. (2017) for a detailed assessment of the species-specific responses of white ash (*Fraxinus americana*) to terrestrial acidification.

Similar numbers and identities of beneficiaries were identified among ecoregions, suggesting that similar users are impacted by terrestrial eutrophication nationally. Indeed, the B7 beneficiaries, which were present in all ecoregions, include any non-consumptive specialized interest group, which can value many if not all FEGS (Landers and Nahlik 2013). These represent a beneficiary set that values the “natural state,” and although this state is difficult to define, they value any FEGS associated with fewer anthropogenic influences. Future efforts will benefit from greater resolution in defining beneficiary subgroups to identify regional differences and other preferences. For example, not all hunters care about the same game animals, and not all Resource-Dependent Businesses rely on the same resources; thus, improved resolution of the beneficiary subgroups will enable more precise linkages of critical load exceedances in a particular place, to the beneficiaries affected.

### SOS implications

There are several lessons that can be extracted from the SOS scoring. First, the observation that the SOS of the weakest link was highly correlated with the SOS of the chain (*r* = 0.85) suggests that more targeted research on these bottlenecks of critical loads could dramatically improve our understanding of links to FEGS. Second, the observation that average SOS metrics tended to be either higher or lower for an ecoregion is deceiving for some ecoregions. Indeed, the moderate average SOS_S_ (0.73) reported for the Eastern Temperate Forests belies great disparity in our confidence in tree critical loads (1.0) as opposed to all other biological indicators (0.33; and lichen not even included). This disparity was also found in the Northwestern Forested Mountains. On the other hand, North American Deserts consistently had some of the lowest SOS scores across all metrics, suggesting that greater research is needed in this ecoregion. Deserts and other aridlands historically have been considered more water limited than nutrient limited (Noy-Meir 1973), and fertilizer comparison studies typically report this (Clark et al. 2007, Hall et al. 2011). However, in wet years arid areas can show strong responses to added nutrients (Rao and Allen 2010, Hall et al. 2011), suggesting that co-varying factors influence aridland sensitivity to terrestrial eutrophication. Third, it is notable how much more is known in the Marine West Coast Forests about the critical loads (average SOS_S_ = 1), compared with the EPF (average SOS_EPF_ = 0.63), suggesting more work is needed to trace these effects through these systems. This ecosystem also had the longest average EPF length (4.6) per chain, highlighting the complexity of the responses that originate with the lichen community.

### Additional uncertainties, limitations, and knowledge gaps

There are several uncertainties and limitations to this assessment, and key knowledge gaps that were identified. At its core, this is a qualitative/semi-quantitative assessment of the impacts from eutrophication on terrestrial ecosystems, constrained by the published literature, the membership of the workshop participants, and the structure of the workshop. Limitations in the literature stem from historical biases in effort regionally, negative publication bias, as well as other factors that limit the comprehensiveness of our assessment. Regional differences contributing to information gaps include a historical emphasis on acidification in the east, eutrophication in the west, an emphasis on lichen in the Pacific North-west, and omission of the Midwest whose landscapes are dominated by human influence, among others. The workshop participants included many of the leading researchers in the United States on terrestrial eutrophication, though not all were present, with the notable omission of researchers active in the alpine meadow environments and eastern forest understory herbs (Gilliam 2006, Bowman et al. 2012). Low representation of many European researchers, though not necessarily a hindrance because of the intended focus on the United States, did remove a vast knowledge pool from potential use that a rich research history in terrestrial eutrophication (Stevens et al. 2004, 2010, Maskell et al. 2010, Dise et al. 2011, Phoenix et al. 2012, Payne et al. 2013, Field et al. 2014). Furthermore, a 3-d workshop to summarize such a vast knowledge space is a difficult task. Finally, it is theoretically possible to more explicitly link FEGS to changes in human well-being than was attempted here, through a deeper dive into that step in the chain (e.g., Fig. 1b, link between FEGS and beneficiaries). However, given that the aim of this effort was to detail the breadth of FEGS affected by changes in biological indicators that are tied to critical loads, and the large number of chains discovered, detailing that step was considered out of scope for this effort. A follow-up effort is underway to attempt to flesh out for a subset of chains how and where changes in select FEGS affect human well-being.

It is important to also note that there are potentially positive impacts from N deposition on terrestrial ecosystems if inputs are low enough. As N is a common limiting nutrient (Vitousek and Howarth 1991), increasing its availability can lead to increased tree aboveground production which may benefit timber producers (Thomas et al. 2010), increases in biodiversity at very low levels of N input that may benefit wildlife viewers (Simkin et al. 2016), and increases in carbon sequestration that may benefit climate regulation (Zaehle et al. 2010b). Follow-on studies could attempt to describe the positive effects from N deposition, so that we could begin to weigh tradeoffs of increasing or decreasing N deposition. However, such an effort is likely to be difficult if not impossible to do objectively. For example, it is not currently possible to weigh an increase in carbon sequestration against the local extirpation of a species for many reasons, including (1) some FEGS has been monetarily valued (i.e., carbon sequestration) while others have not for almost all species, (2) monetary valuation is known to not capture all values, (3) different beneficiaries may value different FEGS, and (4) at different levels. These are merely a few of the challenges that await weighing trade-offs of FEGS, and in quantitatively linking FEGS to beneficiaries. Either way, many of these positive impacts appear to have thresholds where the effect switches to a negative effect, and current levels of deposition are often above these thresholds (Thomas et al. 2010, Pardo et al. 2011a, Sim-kin et al. 2016).

Caveats aside, we believe the output from this effort generally represents the current state of knowledge of the impacts from terrestrial eutrophication via N deposition on U.S. ecosystems and helps identify areas where more study is needed. Key knowledge gaps that could dramatically advance our understanding of terrestrial eutrophication in the United States include but are not limited to:

1. More research on eutrophication is needed in the Eastern Temperate Forests generally, though pioneering studies and recent compilations exist for understory herbs (Gilliam 2006, 2014), and extensive, seminal, research on N impacts to biogeochemical cycling (e.g., Aber et al. 1998) have occurred.
2. Even though there is research on the vulnerability of some tree species in Southern California, more research generally on the sensitivity to N of tree species outside of the Northeast and Mid-Atlantic and on lichen species in the East is needed to balance our understanding of regional taxonomic vulnerabilities.
3. More research is needed to understand why so few biological indicators are affected in some ecoregions (e.g., Marine West Coast Forests and North American Deserts) as opposed to others (e.g., Eastern Temperate Forests and Mediterranean California). Is this a function of lower deposition, weaker N limitation and/or co-limitation with P, publication bias, or more resilient systems?
4. Better refinement and categorization of FEGS and beneficiaries from regionally relevant to locally relevant classes is necessary to explicitly tie critical load exceedances to beneficiaries.
5. Improvements in our confidence of all steps along the chain are needed, from critical load to FEGS to beneficiaries, but especially at the weakest link in the EPF.
6. Extending this STEPS Framework approach to other ecoregions (e.g., Great Plains, Northern Forests) and to finer grained biological indicators, FEGS, beneficiaries, and ecosystems is needed nationally.

## Conclusions

Here, we show that terrestrial eutrophication is a widespread phenomenon across the continental United States and demonstrate the variety of ecosystem services and people affected by this environmental stressor. Because our work did not involve social evaluation of eutrophication-driven FEGS changes, we cannot yet say how large those impacts are in economic or other terms. However, the activity does suggest numerous causal pathways between eutrophication and ecological outcomes that could be economically and socially important. Our assessment is not comprehensive, and thus represents a summary of major impacts considered by experts in the field, and most certainly is an underestimate of the total number of impact pathways nationally. Nevertheless, our assessment was thorough and found that exceedances of 21 N critical loads in five ecoregions affected 582 unique pathways. These exceedances ultimately affected 66 FEGS across a range of categories (21 categories) and 198 regional and FEGS-specific human beneficiaries of various types (16 types). The exact narrative varied widely from place to place, but the general pattern was similar: Species of interest are lost, community composition changes, and secondary effects occur, including changes in fire regimes, runoff and aquifer recharge, carbon sequestration, and habitat of high-value species. These findings underscore the national extent of impacts from terrestrial eutrophication and suggest areas for future research to better enable society to quantify and evaluate the impacts to society from this environmental stressor.

## Supplementary Material

Supplement1

Supplement2

## Figures and Tables

**Table 1.** Overview of biological indicators assessed in this study, the associated ecoregion and ecosystem for which they are reported, the critical load (CL, kg N·ha^−1^·yr^−1^), the strength of science of the critical load (SOS_S_).

Level 1 Ecoregion	Ecosystem	Initial biological indicator	CL	SOS_S_	No. chains	No. FEGS	No. bens	Refs†
Eastern Temperate Forests	hardwood, coniferous forest	Decreased abundance of arbuscular mycorrhizal fungi	<12	0.33	17	2	9	1
		Decreased abundance of ectomycorrhizal fungi	5–10	0.33	17	2	9	1
		Decreased growth of red pine	<4	1.00	25	3	10	2
		Decreased survival of bigtooth aspen	<4	1.00	32	4	11	2
		Decreased survival of scarlet oak	<5	1.00	32	4	11	2
		Decreased survival of trembling aspen	<4	1.00	32	4	11	2
		Increased bacteria-to-fungi ratio	<12	0.33	17	2	9	1
		Increased cover of understory nitrophilic species	<17.5	0.33	18	2	10	1
Marine West Coast Forests	coniferous forest	Decreased lichen biodiversity	2.7–9.2	1.00	62	7	11	1
Mediterranean California	coastal sage scrub	Decreased native mycorrhizal diversity	7.8–9.2	0.67	61	5	10	1
		Increased grass-to-forb ratio and/or increase in total biomass	7.8–10	0.67	38	5	10	1
	grassland	Decreased abundance of arbuscular mycorrhizal fungi	7.8–9.2	0.33	8	1	8	1
		Increased grass-to-forb ratio and/or increase in total biomass	7.8–10	0.33	24	2	9	1
	mixed-conifer forest	Increase in N leaching	17	1.00	2	1	2	1
		Increased bark beetle abundance	39	0.33	34	4	12	1
	serpentine grassland	Increased grass-to-forb ratio and/or increase in total biomass	6	1.00	16	2	9	3
North American Deserts	creosote bush shrubland	Increased grass-to-forb ratio and/or increase in total biomass	3.2–9.3	0.67	31	3	9	3
	pinyon-juniper/Joshua tree woodland	Increased grass-to-forb ratio and/or increase in total biomass	3–6.3	0.67	23	2	8	3
	sagebrush steppe	Increased grass-to-forb ratio and/or increase in total biomass	11	0.33	60	7	12	4
Northwestern Forested Mountains	alpine meadow	Change in herbaceous community composition	3	1.00	7	1	7	5
	mixed-conifer forest	Decreased abundance of ectomycorrhizal fungi	5–10	0.33	26	3	11	1

*Notes:* Also shown are the total number of chains, FEGS, and beneficiaries (Bens) affected for each biological indicator, and associated references (Refs). FEGS, Final Ecosystem Goods and Services.

† References (Refs) are as follows: 1, Pardo et al. (2011a); 2, Thomas et al. (2010); 3, Fenn et al. (2010); 4, Apel et al. (2014); 5, Bowman et al. (2012).

**Table 2.** Numbers of chains, FEGS, and beneficiaries (bens) associated with each initial biological indicator (sorted by number of chains).

Biological indicator	No. chains	No. FEGS	No. bens
Increased grass-to-forb ratio and/or increase in total biomass	192	11	12
Decreased lichen biodiversity	62	7	11
Decreased native mycorrhizal diversity	61	5	10
Decreased abundance of ectomycorrhizal fungi	43	3	12
Increased bark beetle abundance	34	4	12
Decreased survival of bigtooth aspen	32	4	11
Decreased survival of scarlet oak	32	4	11
Decreased survival of trembling aspen	32	4	11
Decreased abundance of arbuscular mycorrhizal fungi	25	3	10
Decreased growth of red pine	25	3	10
Increased cover of understory nitrophilic species	18	2	10
Increased bacteria-to-fungi ratio	17	2	9
Change in herbaceous community composition	7	1	7
Increase in N leaching	2	1	2

*Note:* FEGS, Final Ecosystem Goods and Services.

**Table 3.** Number of chains and beneficiaries (bens), and the identity of the beneficiaries, associated with a change in ecological endpoint.

Response in ecological endpoint	No. chains	No. bens	Ben identity
Change in forest composition	79	8	B7, TFO
Change in herbaceous community composition	7	7	B7
Decreased abundance and/or diversity of birds and mammals	98	9	B7, H, RDB
Decreased abundance of truffles	1	1	FP&G
Decreased aquifer recharge	1	1	WS
Decreased Bay checkerspot butterfly abundance	7	7	B7
Decreased diluting power as supply of drinking water	2	2	RDB, WS
Decreased flying squirrel abundance	7	7	B7
Decreased forest productivity/C sequestration	87	9	B7, AH, TFO
Decreased Joshua tree cover	7	7	B7
Decreased lichen presence	14	7	B7
Decreased native plant diversity/species composition (e.g., T&E Species)	116	10	B7, LG, RPO, RDB
Decreased pollinator presence	10	10	B7, FE, FP&G, RDB
Decreased quality of California gnatcatcher habitat	21	7	B7
Decreased quality of fall foliage	24	8	B7, RDB
Decreased sage grouse abundance	8	8	B7, H
Decreased shrub cover	29	8	B7, LG
Decreased spotted owl abundance	7	7	B7
Decreased stream levels	1	1	LG
Increased fire frequency	49	9	B7, RPO, RDB
Increased pinyon pine mortality	7	7	B7

*Note:* B7, B7 beneficiaries; TFO, timber, fiber, and ornamental extractors; H, hunters; RDB, Resource-Dependent businesses; FP&G, food pickers and gatherers; WS, water subsisters; AH, all humans; LG, livestock grazers; RPO, residential property owners; FE, food extractors.

**Table 4.** Numbers of chains affecting each beneficiary group.

Beneficiaries	No. chains
Artists†	72
Educators and Students†	72
Experiencers and Viewers†	72
People Who Care (Existence)†	72
People Who Care (Option/Bequest)†	72
Researchers†	72
Spiritual and Ceremonial Participants and Participants of Celebration†	72
Resource-Dependent Businesses	22
Timber, Fiber, and Ornamental Extractors	16
Hunters	13
All Humans	10
Residential Property Owners	7
Livestock Grazers	5
Food Pickers and Gatherers	2
Water Subsisters	2
Food Extractors	1

† Member of the B7 beneficiary group.

**Table 5.** Average strength of science (SOS) for critical loads (S), EPFs, WLs, and the Chain among ecoregions.

	SOS score			
	
Ecoregion	S	EPF	WL	Chain
Eastern Temperate Forests	0.77	0.81	0.77	0.80
Marine West Coast Forests	1.00	0.63	0.74	0.71
Mediterranean California	0.71	0.77	0.69	0.75
North American Deserts	0.47	0.70	0.47	0.63
Northwestern Forested Mountains	0.46	0.83	0.46	0.73

*Notes:* EPF, Ecological Production Function; WL, weakest link. Averages are calculated based on the 76 unique EPFs identified and therefore are not weighted by the number of beneficiaries associated with each ecological endpoint.

## Box 1 Tree species decline, fall colors, and mycorrhizal associations

Eastern tree species are critical for providing many Final Ecosystem Goods and Services including timber production, clean air and water, beautiful vistas, and habitat for game and non-game animal species. Changes in these ecosystem goods and services driven by N eutrophication could affect many beneficiaries, including timber producers, hunters, tourism-related businesses, and recreational users of all types.

Several eastern tree species are reported to be negatively affected by N deposition (Fig. 8), including red pine (*Pinus resinosa*), bigtooth aspen (*Populus grandidentata*), trembling aspen (*Populus tremuloides*), and scarlet oak (*Quercus coccinea*; Thomas et al. 2010), and the northern areas of this ecoregion have very low reported critical loads of nutrient N (Duarte et al. 2013). Thomas et al. (2010) reported that with N deposition above 3–5 kg ha^−1^ yr^−1^, survival and/or growth for all four species decreased. Each of these species provides a variety of ecosystem services. Red pine, bigtooth aspen, and trembling aspen are all important sources for wood products, especially trembling aspen that is used for particle boards, matchsticks, and tongue depressors (USFS 2016). All four tree species are important for many wildlife species, including wild turkeys that feed on scarlet oak acorns, rabbits that rely on trembling aspen year round for food, white-tailed deer that browse saplings of all four species, and many species of game and non-game birds that utilize these tree species for nesting sites and food (USFS 2016). The mixture of these deciduous and evergreen species across the landscape also contributes to the fall foliage colors and associated ecotourism. These ecosystem services affect many beneficiaries, including timber producers, hospitals, hunters, and recreational users to name a few.

It is difficult to confidently isolate these impacts to N or S deposition alone since these pollutants co-occur across the landscape. However, the significant decrease in especially S deposition since peaks of the 1970s and 1980s still yielded negative responses in Thomas et al. (2010), and more recent analyses by Horn et al. (*unpublished manuscript*) where they explicitly controlled for S deposition indicate that N deposition can have negative effects in isolation. Whether this is a eutrophication effect from faster-growing neighbors outcompeting some species, or an acidification effect from N, remains to be resolved.

It has been hypothesized (Thomas et al. 2010) that tree species with ectomycorrhizal associations tend to be negatively affected by increasing N deposition, while those with arbuscular associations tend to be positively affected. This is thought to be because arbuscular mycorrhizae, unlike ectomycorrhizal fungi, do not produce enzymes that break down soil organic N. Thus, trees with arbuscular associations may be more likely to benefit from increased soil N availability from deposition. Lilleskov et al. (2008) found that ectomycorrhizal fungi in northern forests can be negatively affected by N deposition above 5–7 kg ha^−1^ yr^−1^, for a range of tree species. Given the ubiquity of mycorrhizal associations in forests, and their importance to tree health as well as carbon and N cycling, impacts on these life forms could have large effects on forest structure and function.

As the tree communities shift from terrestrial eutrophication, not only will the resulting fall color display will be affected, but many commercially important wood products and the natural habitat for several species of birds and mammals will be altered. These changes in turn will affect many groups of beneficiaries that enjoy and depend upon the forests of the eastern United States.

## Box 2 Network of effects from losses of horsehair lichen in the Northwest

Here, we illustrate some ecosystem services and human end users connected with horsehair lichens (*Bryoria* spp.). These dark brown, arboreal lichens are common inhabitants of coniferous forests of the northwestern mountains and boreal regions of North America, attaining greatest biomass in old-growth forests (Brodo et al. 2001). They are highly sensitive to nutrient N and acidic deposition, declining in detection probability with 2.4 kg ha^−1^ yr^−1^ of N (Fig. 9) and 0.75 kg ha^−1^ yr^−1^ of S deposition or less (McCune and Geiser 2009, Root et al. 2015).

The horsehair lichens perform a range of critical functions in different habitats. In the northern Rocky Mountains, *Bryoria* species (e.g., *Bryoria fremontii*) can comprise 93% of the December to April diet for the northern flying squirrel (Maser et al. 1985) and 96% of its nesting material (Hayward and Rosentreter 1994). They also constitute the bulk of winter forage for the endangered woodland caribou (Rominger et al. 2000). Thus, winter survival of both of these species depends on the availability of these lichens. Along with wood rats and red-backed voles, the northern flying squirrel is a primary prey of the northern spotted owl, an endangered species of the north-western U.S. old-growth forests (Maser et al. 1978). Many other species of bird also utilize lichen directly. Over 100 bird species use lichens as nest construction materials, foraging and breeding habitat, and/or camouflage (Rodewald 2016). Horsehair lichens are of notable use as nest lining materials by songbirds such as Swainson’s thrush, the warbling vireo, and endangered species including Bachman’s warbler, the varied thrush, and the yellow-throated vireo.

Horsehair lichens have been extensively used by indigenous peoples of North America (Craw-ford 2015). For example, horsehair lichens have been used by the Sahaptin of Oregon and Washington for millennia as a poultice for arthritis, by the Nimi’ipuu of Montana as a cure for upset stomach, indigestion, and diarrhea, and by the Okanagan of British Columbia in syrups and salves to protect newborn babies from infection, among many other uses (Crawford 2015). The 1000+ unique secondary compounds produced by lichens, including the horsehair lichens, are being actively tested for their anti-bacterial, anti-cancer, and antioxidant properties. Such potential pharmaceutical uses are the basis for a new and growing area of research (Rancovíc 2015).

Thus, protection of horsehair lichen supports a wide range of ecosystem services. These in turn benefit many groups of people, including recreational birders, wildlife enthusiasts, traditional cultures, tourism-dependent businesses, beneficiaries of pharmaceutical research, and future generations for whom endangered species are protected.

## Box 3 Coastal sage scrub, invasive grasses, and fire

Coastal sage scrub (CSS) is a semi-deciduous shrubland that occurs in the Mediterranean-type climate of southern and central coastal California, extending southward to Baja California, Mexico (Fig. 10a). The plant communities within the CSS vegetation type, which encompass over 6300 km^2^ of habitat in California (Fenn et al. 2010), are especially diverse, including many native forbs. A high proportion of these forbs are annuals, some of which, along with many species of insects, are species of concern under the Endangered Species Act (Hernández et al. 2016) including San Diego button-celery (*Eryngium aristulatum* var. *parishii*) and Slender-horned spineflower (*Dodecahema leptocerus*). Coastal sage scrub also provides habitat for endangered wildlife, such as the California gnatcatcher, and game animals, such as the California quail and mule deer. These areas are highly impacted by N pollution emanating out of the Los Angeles air basin.

Studies along anthropogenic N deposition gradients and in experimentally fertilized plots have shown a variety of impacts from N enrichment in this ecosystem. Responses include increases in exotic invasive annual grasses, loss of native shrub and forb cover, reduced diversity of native annual forbs and arbuscular mycorrhizal fungi, and elevated N mineralization. Plants in this low-productivity ecosystem respond quickly to N; thus, changes in species abundances are excellent indicators of ecosystem response to N pollution (Fenn et al. 2010). The estimated N critical load for increases in exotic grass cover and decreases in native plant species diversity is 7.8–10 kg N ha^−1^ yr^−1^, and for decreases in arbuscular mycorrhizal spore density and root infection is 7.8–9.2 kg N ha^−1^ yr^−1^ (Egerton-Warburton and Allen 2000, Pardo et al. 2011b). One challenge in setting critical loads for CSS and other historically polluted areas is that the lowest deposition values measured in the studied area (6.6–8.7 kg ha^−1^ yr^−1^) is already significantly elevated above pre-industrial background deposition (<2 kg ha^−1^ yr^−1^, Pardo et al. 2011b). Therefore, the critical load values given above should be considered as upper limit values.

Thus, CSS vegetation in southern California has been rapidly converting to exotic annual grass-land in the past 30–40 yr (Talluto and Suding 2008). The conversion of CSS to grassland is likely caused by a combination of elevated N deposition, that promotes increased grass biomass, and frequent fire, which in turn prevents re-establishment of native shrubs and forbs (Fig. 10b). As native forbs and shrubs disappear, so does the habitat for sensitive species native to this area. This increase in fire frequency acts as an intermediate and final FEGS, as the fire itself directly impacts residential property owners, while the landscape changes induced by fire affect the vegetation and wildlife communities that are valued by many beneficiaries, including tourism-dependent businesses, recreational birders, and people who value undisturbed habitats of the Mediterranean California Ecoregion.
