# Supplementary material for: Nitrogen-induced terrestrial eutrophication: cascading effects and impacts on ecosystem services
Source: Ecosphere. Author manuscript; Available in PMC 2024 Dec 12. (PMC11636942; doi:10.1002/ecs2.1877)
Supplement: Supplement1 [file NIHMS1993687-supplement-Supplement1.zip › Clark_ECS16-0532R1_Metadata_S1.pdf]

A framework to quantify the strength of ecological links between an environmental stressor and final ecosystem services

Michael D. Bell, Jennifer Phelan, Tamara F. Blett, Dixon Landers, Amanda M. Nahlik, George Van Houtven, Christine Davis, Christopher M. Clark, Julie Hewitt

Ecosphere

Relationships linking a change in a biological indicators of atmospheric deposition to final ecosystem goods and services.

This data is a compilation of four data sets from the subject papers that make up this the special section "Air Quality and Ecosystem Services". The authors of each section are:

Aquatic Acidification: Claire O'Dea[1,6], Sarah Anderson[2], Timothy Sullivan[3], Dixon Landers[4], and Frank Casey[5]

[1]United States Department of Agriculture Forest Service, Washington, DC 20250 USA
[2]Washington State University, Pullman, WA 99164 USA
[3]E&S Environmental Chemistry, Inc., Corvallis, OR 97339 USA
[4]U.S. Environmental Protection Agency, Corvallis, OR 97333 USA
[5]U.S. Geological Survey, Washington, DC 20230 USA

Aquatic Eutrophication: Charles Rhodes,[1†] Andrew Bingham,[2] Andrea M. Heard,[3] Julie Hewitt,[4] Jason Lynch,[5] and Randall Waite,[6] Michael D. Bell[2]

[1]Oak Ridge Institute for Science and Education post-doctoral fellow, participating at U.S. Environmental Protection Agency, Office of Water, and Office of Research and Development, Washington, DC, USA 20460
[2]National Park Service, Air Resources Division, Denver, CO, USA 25287
[3]National Park Service, Sierra Nevada Network, Three Rivers, CA, USA 93271
[4]U.S. Environmental Protection Agency, Office of Water, Washington, DC, USA 20460
[5]U.S. Environmental Protection Agency, Office of Air and Radiation, Washington, DC, USA 20460

Terrestrial Acidification: Irina C. Irvine[1*], Tara Greaver[2], Jennifer Phelan[3], Robert D. Sabo[4], and George Van Houtven[3]

[1]Santa Monica Mountains National Recreation Area, Division of Planning Science and Resource Management, US National Park Service, Thousand Oaks, CA 91360
[2]Office of Research and Development, National Center for Environmental Assessment, US Environmental Protection Agency, Research Triangle Park, NC 27709
[3]RTI International, Research Triangle Park, NC 27709
[4]Currently an Oak Ridge Institute for Science and Education (ORISE) Fellow, Office of Research and Development, National Climate Assessment Global Change Impacts and Adaptations, Environmental Protection Agency, Crystal City, VA 22202

Terrestrial Eutrophication: Christopher M. Clark[1], Michael D. Bell[2], James W. Boyd[3], Jana Compton[4], Eric Davidson[5], Christine Davis[6], Mark Fenn[7], Linda Geiser[8], Laurence Jones[9], Tamara F. Blett[2]
[1]US EPA, Office of Research and Development, National Center for Environmental Assessment, Washington, DC, 20460
[2]Air Resources Division, National Park Service, Lakewood, Colorado, 80225
[3]Resources for the Future, Washington, DC, 20036
[4]Office of Research and Development, Western Ecology Division, Corvallis, OR, 97333
[5]Appalachian Laboratory, University of Maryland Center for Environmental Science, Frostberg, MD, 21532
[6]US EPA, Office of Air and Radiation, Office of Air Quality Planning and Standards, Research Triangle Park, NC, 27709
[7]USDA Forest Service, Pacific Southwest Research Station, Riverside, CA, 92607
[8]USDA Forest Service, Washington Office-Water Wildlife Fish Air and Rare Plants, Washington, DC, 20250
[9]Centre for Ecology and Hydrology, Environment Centre Wales, Deiniol Road Bangor, LL57 2UW, United Kingdom

DataS1.xlsx

'DataS1.xlsx' lists all of the chains developed to link change in a biological indicator due to exceedance of a critical load to an ecological component that is a final ecosystem service. There are 1104 chains from the four ecological effects; 208 from Aquatic Acidification, 154 from Aquatic Eutrophication, 160 from Terrestrial Acidification, and 582 from Terrestrial Eutrophication. The table below provides descriptions of each of the columns.

This data set can be used to sort by your interested field to evaluate the relationships among the chains and the beneficiary categories. Identifying places within the table where additional stressors are acting will allow us to expand the analysis to evaluate where synergistic effects may occur. It can also allow a user to sort by the type of beneficiary they are interested in to determine if the indicators exist in their area and where management actions can take place.

Module	Column Header	Description
	Ecological Effect	The broad consequence of nitrogen and/or acid deposition on an ecosystem to be evaluated
Site Information	Region	Regional designation of where critical load is applicable
Site Information	Ecosystem	Ecosystem type in which critical load is applicable based on EPA designated Ecosystem Level I,II, and III

Stressor	Chemical Criterion	Soil (e.g., soil solution Bc/Al ratio or % base saturation) or surface water chemistry (e.g., ANC or pH) that links the critical load of atmospheric deposition to the biological receptor of interest. Deposition alters the chemical criterion, and the critical load of a system is the point where deposition levels result in the chemical criterion being equal to the critical limit. Chemical criterion are commonly used in models to estimate aquatic and terrestrial critical loads of acidity. If species change has been measured to correlate with a deposition gradient, the deposition itself is the chemical criterion.
Stressor	Chemical Threshold	The threshold or value of the chemical criterion beyond which the biological receptor of interest is negatively impacted (e.g., 20% base saturation, ANC of 50 µeq/L). The critical load of a system is the point where deposition levels result in the chemical criterion being equal to the critical limit.
Stressor	Biological Indicator	A biological species or group of species whose function, population, or status can reveal the qualitative status of the environment, and can therefore be used to monitor the health of an environment or ecosystem.
Stressor	Critical Load (CL; in kg/ha)	The amount of atmospheric deposition above which adverse effects begin to affect the biological indicator
Stressor	Low	If the critical load has been established as a range; the low value of the range.
Stressor	High	If the critical load has been established as a range; the high value of the range.
Stressor	$SOS_{Stressor}$	Strength of science of the calculated critical load
Stressor	CL Reference	Scientific publication used to verify $SOS_{Stressor}$
Stressor	Change in Biological Indicator (Component #1)	The effect on the ecosystem component due to exceedance of the critical load or the chemical criterion. The change in biological indicator acts as the first component of the Ecological Production Function
Ecological Production Function	SOS – Effect #	Strength of Science explaining how the the proceeding ecological components causes a change in the proceeding ecological component. Values are High = 1, Medium = 0.67, Low= 0.33
Ecological Production Function	Ref #	Scientific publication(s) linking the change of $Component_i$ to $Component_{i+1}$
Ecological Production Function	Component #	Ecosystem component influenced by a change in the ecosystem component before it.

Ecological Production Function	Ecological Endpoint	Ecological component that provides a service or is valued by humans. This is the same as the last link in the chain, but allows for chains to be categorized due to different chain lengths.
Final Ecosystem Services	Beneficiary sub-classes (direct FEGS user)	FEGS-CS Beneficiary Sub-Class: beneficiary that directly uses the last thing in environment directly used by humans.
Final Ecosystem Services	Beneficiary Classes	FEGS-CS Beneficiary Class
Final Ecosystem Services	FEGS Metric	The measurement taken to classify the Ecological Endpoint in a form relevant to the beneficiary sub-class
Strength of Science	EPF Length	Number of links within the Ecological Production Function
Strength of Science	SOS_{EPF}	Calculation of EPF using number of components in the chain and the average score of the chain; Equation 1
Strength of Science	SOS_{WL}	Value of the lowest SOS_{Effect} or $SOS_{Stressor}$ score in the chain
Strength of Science	SOS_{CHAIN}	Calculation using the SOS_{Effect} and $SOS_{Stressor}$ scores to define the confidence in scientific knowledge of the relationships defined; Equation 2
