# Supplementary material for: Nitrogen-induced terrestrial eutrophication: cascading effects and impacts on ecosystem services
Source: Ecosphere. Author manuscript; Available in PMC 2024 Dec 12. (PMC11636942; doi:10.1002/ecs2.1877)
Supplement: Supplement2 [file NIHMS1993687-supplement-Supplement2.pdf]

Appendix S1

Table S1. Modified FEGS-CS matrix used for the workshop (Landers and Nahlik 2013).

Beneficiary Categories and Sub-Categories	FINAL ECOSYSTEM GOODS AND SERVICE (FEGS) IDENTIFIED FOR EACH ENVIRONMENTAL SUB-critical loadASS							
	**Rivers and Streams**	**Wetlands**	**Lakes and Ponds**	**Forests**	**Grasslands**	**Scrubland/ Shrubland**	**Tundra**	**Ice and Snow**
Agriculture								
Aquaculturists	presence of the environment, water	presence of the environment, water	presence of the environment, water					
Livestock Grazers	water, flora		water	water, flora, open space	flora, open space	flora, open space		
Foresters				soil, open space				
Commerical/Industrial								
Food Extractors	flora, fauna	flora, fauna	flora, fauna	flora, fauna, fungi	flora, fauna, fungi	flora	flora, fauna	
Timber, Fiber, Ornamental Extractors	fiber, natural materials	timber, fiber, natural materials	natural materials	fiber, natural materials, timber	fiber, natural materials	timber, fiber, natural materials	fiber, natural materials	
Resource Dependent Businesses	presence of the environment							
Pharmaceutical and Food Supplement Suppliers	flora, fauna	flora, fauna	flora, fauna	flora, fauna, fungi	flora, fauna, fungi	flora		
Subsistence								
Water Subsisters	water		water					water
Food Subsisters	flora, fauna	flora, fauna	flora, fauna	flora, fauna, fungi	flora, fauna, fungi	flora, fauna	flora, fauna	fauna
Timber, Fiber, and Fur/Hide Subsisters	fiber, fauna	timber, fiber, natural materials	fiber, fauna	timber, fiber, fauna	timber, fiber, fauna	timber, fiber, fauna	timber, fiber, fauna	fauna
Recreational								
Experiencers and Viewers	presence of the environment, viewscapes, flora, fauna, sounds and scents	presence of the environment, viewscapes, flora, fauna, sounds and scents	presence of the environment, viewscapes, flora, fauna, sounds and scents	presence of the environment, viewscapes, flora, fauna, fungi, sounds and scents	presence of the environment, viewscapes, flora, fauna, fungi, sounds and scents	presence of the environment, viewscapes, flora, fauna, fungi, sounds and scents	presence of the environment, viewscapes, flora, fauna, sounds and scents	presence of the environment, viewscapes, fauna, sounds and scents
Food Pickers and Gatherers	flora, fauna	flora, fauna	flora, fauna	flora, fauna, fungi	flora, fauna, fungi	flora, fauna	flora	
Hunters	fauna	fauna	fauna	fauna	fauna	fauna	fauna	fauna
Anglers	fish	fish	fish					
Waders, Swimmers, and Divers	presence of the environment	presence of the environment	presence of the environment					
Boaters	presence of the environment, water	presence of the environment, water	presence of the environment, water					
Inspirational								
Spritual and Ceremonial Participants and Participants of Celebration	presence of the environment							
Aritsts	presence of the environment, viewscapes, sounds and scents, natural materials	presence of the environment, viewscapes, sounds and scents, natural materials	presence of the environment, viewscapes, sounds and scents, natural materials	presence of the environment, viewscapes, sounds and scents, natural materials	presence of the environment, viewscapes, sounds and scents, natural materials	presence of the environment, viewscapes, sounds and scents, natural materials	presence of the environment, viewscapes, sounds and scents, natural materials	presence of the environment, viewscapes, sounds and scents, natural materials
Learning								
Eduators and Students	presence of the environment							
Researchers	presence of the environment							
Non-Use								

People Who Care (Existence)	presence of the environment
People Who Care (Option/Bequest)	presence of the environment

Literature Cited for Supporting Information

Landers, D. H., and A. M. Nahlik. 2013. Final ecosystem goods and services classification system (FEGS-CS). Report number EPA/600/R-13/ORD-004914. United States Environmental Protection Agency, Washington, DC.
